# Rhamnosyltransferases involved in the biosynthesis of flavone rutinosides in *Chrysanthemum* species

**DOI:** 10.1093/plphys/kiac371

**Published:** 2022-08-10

**Authors:** Qing-Wen Wu, Min Wei, Ling-Fang Feng, Li Ding, Wu-Ke Wei, Jin-Fen Yang, Xiao-Jing Lin, Hui-Lin Liang, Ruo-Ting Zhan, Dong-Ming Ma

**Affiliations:** Research Center of Chinese Herbal Resource Science and Engineering, Guangzhou University of Chinese Medicine, Guangzhou 510006, China; Key Laboratory of Chinese Medicinal Resource from Lingnan, Guangzhou University of Chinese Medicine, Guangzhou 510006, China; School of Pharmaceutical Sciences, Guangzhou University of Chinese Medicine, Guangzhou 510006, China; China Resources Sanjiu Medical & Pharmaceutical Co., Ltd, Shenzhen 518110, China; Research Center of Chinese Herbal Resource Science and Engineering, Guangzhou University of Chinese Medicine, Guangzhou 510006, China; Key Laboratory of Chinese Medicinal Resource from Lingnan, Guangzhou University of Chinese Medicine, Guangzhou 510006, China; School of Pharmaceutical Sciences, Guangzhou University of Chinese Medicine, Guangzhou 510006, China; Research Center of Chinese Herbal Resource Science and Engineering, Guangzhou University of Chinese Medicine, Guangzhou 510006, China; Key Laboratory of Chinese Medicinal Resource from Lingnan, Guangzhou University of Chinese Medicine, Guangzhou 510006, China; School of Pharmaceutical Sciences, Guangzhou University of Chinese Medicine, Guangzhou 510006, China; Research Center of Chinese Herbal Resource Science and Engineering, Guangzhou University of Chinese Medicine, Guangzhou 510006, China; Key Laboratory of Chinese Medicinal Resource from Lingnan, Guangzhou University of Chinese Medicine, Guangzhou 510006, China; School of Pharmaceutical Sciences, Guangzhou University of Chinese Medicine, Guangzhou 510006, China; Research Center of Chinese Herbal Resource Science and Engineering, Guangzhou University of Chinese Medicine, Guangzhou 510006, China; Key Laboratory of Chinese Medicinal Resource from Lingnan, Guangzhou University of Chinese Medicine, Guangzhou 510006, China; School of Pharmaceutical Sciences, Guangzhou University of Chinese Medicine, Guangzhou 510006, China; Research Center of Chinese Herbal Resource Science and Engineering, Guangzhou University of Chinese Medicine, Guangzhou 510006, China; Key Laboratory of Chinese Medicinal Resource from Lingnan, Guangzhou University of Chinese Medicine, Guangzhou 510006, China; School of Pharmaceutical Sciences, Guangzhou University of Chinese Medicine, Guangzhou 510006, China; Research Center of Chinese Herbal Resource Science and Engineering, Guangzhou University of Chinese Medicine, Guangzhou 510006, China; Key Laboratory of Chinese Medicinal Resource from Lingnan, Guangzhou University of Chinese Medicine, Guangzhou 510006, China; School of Pharmaceutical Sciences, Guangzhou University of Chinese Medicine, Guangzhou 510006, China; Research Center of Chinese Herbal Resource Science and Engineering, Guangzhou University of Chinese Medicine, Guangzhou 510006, China; Key Laboratory of Chinese Medicinal Resource from Lingnan, Guangzhou University of Chinese Medicine, Guangzhou 510006, China; School of Pharmaceutical Sciences, Guangzhou University of Chinese Medicine, Guangzhou 510006, China; Research Center of Chinese Herbal Resource Science and Engineering, Guangzhou University of Chinese Medicine, Guangzhou 510006, China; Key Laboratory of Chinese Medicinal Resource from Lingnan, Guangzhou University of Chinese Medicine, Guangzhou 510006, China; School of Pharmaceutical Sciences, Guangzhou University of Chinese Medicine, Guangzhou 510006, China

## Abstract

Linarin (acacetin-7-*O*-rutinoside), isorhoifolin (apigenin-7-*O*-rutinoside), and diosmin (diosmetin-7-*O*-rutinoside) are chemically and structurally similar flavone rutinoside (FR) compounds found in *Chrysanthemum* L. (Anthemideae, Asteraceae) plants. However, their biosynthetic pathways remain largely unknown. In this study, we cloned and compared FRs and genes encoding rhamnosyltransferases (RhaTs) among eight accessions of *Chrysanthemum* polyploids. We also biochemically characterized RhaTs of *Chrysanthemum* plants and *Citrus* (*Citrus sinensis* and *Citrus maxima*). RhaTs from these two genera are substrate-promiscuous enzymes catalyzing the rhamnosylation of flavones, flavanones, and flavonols. Substrate specificity analysis revealed that *Chrysanthemum* 1,6RhaTs preferred flavone glucosides (e.g. acacetin-7-*O*-glucoside), whereas Cs1,6RhaT preferred flavanone glucosides. The nonsynonymous substitutions of RhaTs found in some cytotypes of diploids resulted in the loss of catalytic function. Phylogenetic analysis and specialized pathways responsible for the biosynthesis of major flavonoids in *Chrysanthemum* and *Citrus* revealed that rhamnosylation activity might share a common evolutionary origin. Overexpression of RhaT in hairy roots resulted in 13-, 2-, and 5-fold increases in linarin, isorhoifolin, and diosmin contents, respectively, indicating that RhaT is mainly involved in the biosynthesis of linarin. Our findings not only suggest that the substrate promiscuity of RhaTs contributes to the diversity of FRs in *Chrysanthemum* species but also shed light on the evolution of flavone and flavanone rutinosides in distant taxa.

## Teaser

The discovery of rhamnosyltransferases in eight accessions of *Chrysanthemum* species contributes to the biosynthesis of flavone rutinosides and evolution of glycosyltransferases in plants.

## Introduction

Polyploidy is a source of evolutionary innovation and species diversification (Van de Peer et al., 2021). Both polyploidy formation and hybridization are ubiquitous in the genus *Chrysanthemum* (Asteraceae), which comprises approximately 40 species exhibiting varying degrees of polyploidy, from diploid to decaploid, with 9 chromosomes as the basal unit (Liu et al., 2012). *Chrysanthemum morifolium* is used as an ornamental and medicinal plant worldwide and is a hexaploid species (2*n *=* *6*x *=* *54) (Drewlow et al., 1973; van Geest et al., 2017; Won et al., 2017). The diploid species *Chrysanthemum indicum* and *Chrysanthemum nankingense* are native to China, and both these species contribute to the origin of polyploidy in *C. morifolium* (Yang et al., 2006; Wang et al., 2014; Won et al., 2017; Song et al., 2018). The diploid form of *C. indicum* is found only in central and northern China, whereas the presence of its tetraploid form has expanded southward and appears to be widespread (Li et al., 2014). Although the morphological and physiological characteristics of *Chrysanthemum* polyploids have been studied extensively (Su et al., 2019), inadequate information is available regarding specialized metabolic pathways, especially those of pharmaceutically active flavone diglycosides.

Linarin, a glycosylated flavone, is identified in over 30 plant species from 13 families, and the majority of these species belong to the family Asteraceae (Mottaghipisheh et al., 2021). Isorhoifolin was originally isolated from the leaves of *Parquetina nigrescens* (Ogundaini and Okafor, 1987). Diosmin is a naturally occurring flavone glycoside that was isolated in 1925 from *Scrophularia nodosa* and introduced as a therapeutic agent in 1969 (Bogucka-Kocka et al., 2013). The aforementioned flavone diglycosides have been isolated from *C. indicum*, *C. nankingense*, and *C. morifolium* (Xie et al., 2009; Han et al., 2015; Song et al., 2018; Chen et al., 2019; Mottaghipisheh et al., 2021). Diosmin is used in pharmaceutical formulations, such as Daflon, Diosed, and Dioven, for the treatment of venous diseases (Gerges et al., 2022). Diosmin is obtained by the dehydrogenation of the flavanone glycoside hesperidin through chemical semisynthesis. Linarin and isorhoifolin, which are chemically and structurally similar, exhibit many potential therapeutic properties, such as those against Alzheimer disease, osteoporosis, and diabetes (Baris et al., 2011; Bansal et al., 2012; Albohy et al., 2020; Mottaghipisheh et al., 2021). The biosynthetic pathway of these active natural products remains largely unknown.

Among the aforementioned three rutinosides, rhamnose is attached through the hydroxyl group at the C6 position of the glucose moiety. Thus, the key step in flavone diglycoside biosynthesis is assumed to be catalyzed by 1,6-rhamnosyltransferase (1,6RhaT). UDP-glycosyltransferases (UGTs) catalyze the transfer of sugar residues from active sugars, such as UDP-glucose, to acceptor substrates. Many UGTs involved in flavonoid biosynthesis have been identified, including flavonoid-7-*O*-glucosyltransferases (7GlcTs) responsible for the formation of a glycosidic bond at position 7 of the flavonoid skeleton, and the sugar moiety has been identified and characterized (McIntosh et al., 1990; Durren and McIntosh, 1999; Kim et al., 2006; Li et al., 2014, 2020; Funaki et al., 2015). In addition to position 7, glycosylation occurs on the 5-hydroxy group on the A-ring and the 3-hydroxy group on the C-ring. By contrast, fewer UGTs are responsible for the formation of a sugar–sugar bond in flavonoid glucosides. Cs1,6RhaT (*Citrus sinensis*) catalyzes the rhamnosylation of flavonoid-7-*O*-glucose substrates at position 6 of the glucose moiety. Cs1,6RhaT is a branch-forming glycosyltransferase involved in the biosynthesis of tasteless flavanone rutinosides commonly observed in non-bitter citrus species (Frydman et al., 2013). Fe1,6RhaT (*Fagopyrum esculentum*) and Gm1,6RhaT (*Glycine max*) are involved in the biosynthesis of flavonol-3-*O*-rutinosides (Rojas Rodas et al., 2014; Koja et al., 2018). In addition, Ph1,6RhaT (*Petunia × hybrid*) controls the rhamnosylation of reddish anthocyanin-3-*O*-glucosides; this is the first step in a series of modifications that yield magenta or blue/purple anthocyanins (Kroon et al., 1994). Branch-forming UGTs involved in the modification of flavone compounds in *Chrysanthemum* species remain to be identified.

This study compared major flavone glucosides and flavone rutinosides (FR) present in tetraploid *C. indicum* and its progenitor species, including diploid *C. indicum* and *C. nankingense*. Gene mining and biochemical characterization of 1,6RhaTs present in *Chrysanthemum* polyploids were performed. Moreover, links between gene evolution and function were evaluated. Our results revealed that 1,6RhaTs in diploid and tetraploid *Chrysanthemum* species catalyzes three flavone-7-*O*-glucosides (FGs) to produce the corresponding flavone-7-*O*-rutinosides (FRs). These results provide biochemical evidence for variations in FRs during *Chrysanthemum* evolution. Our findings can facilitate the use of synthetic biology for producing high-value metabolites.

## Results

### Polyploidy, morphological variation, and geographical distribution of species in the *Chrysanthemum* complex

**Figure 1:**
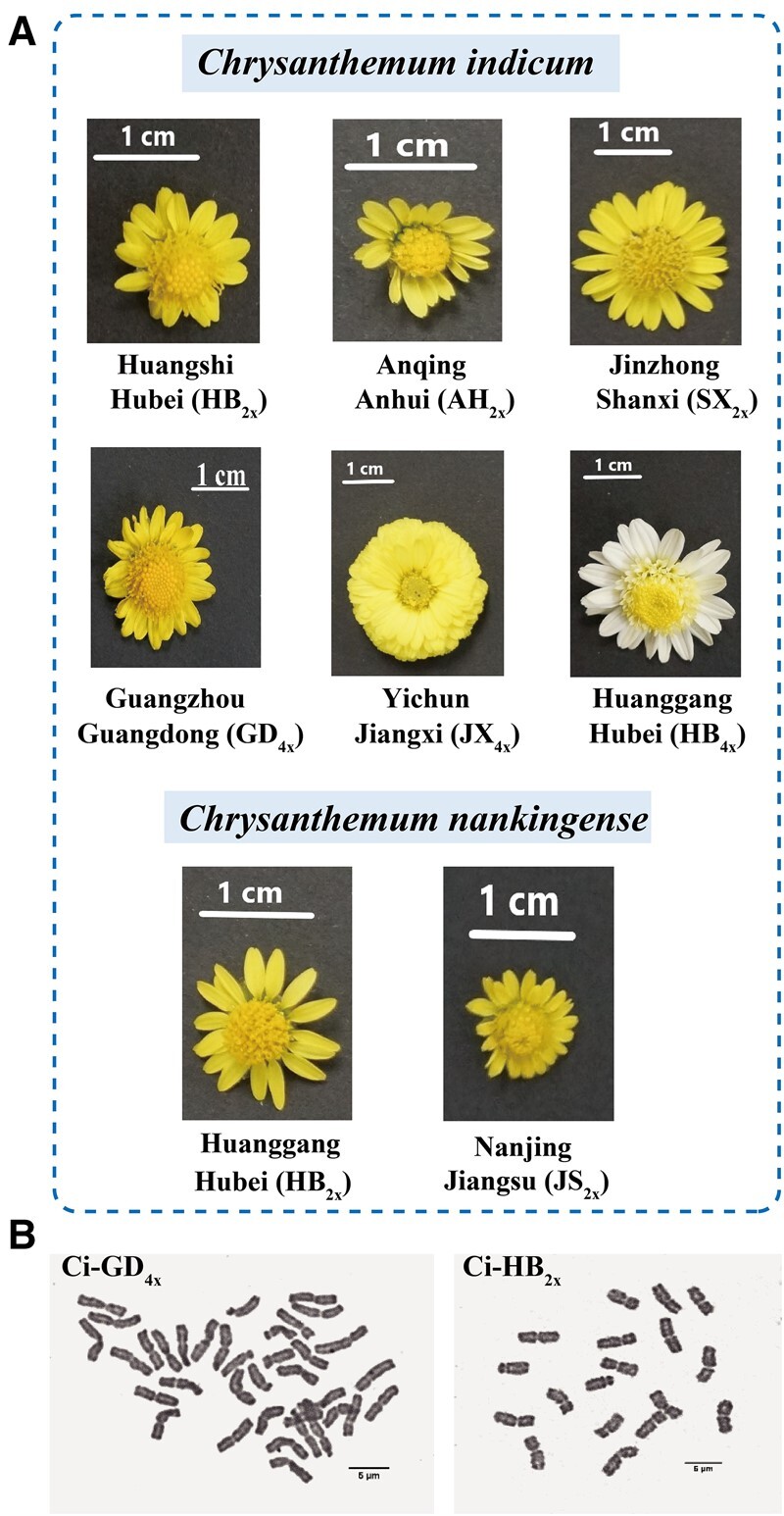
Geographical distribution of and morphological variations among diploid *C. indicum*, tetraploid *C. indicum*, and diploid *C. nankingense*. A, *Chrysanthemum indicum* population from Huangshi, Hubei Province; Anqing, Anhui Province; Jinzhong, Shanxi Province; Guangzhou, Guangdong Province; Yichun, Jiangxi Province; and Huanggang, Hubei Province; *C. nankingense* population from Huanggang, Hubei Province and Nanjing, Jiangsu Province. B, Geographical distribution of the eight accessions of *Chrysanthemum* in China. C, Somatic chromosomes at the mitotic metaphase of the representative diploid and tetraploid *C. indicum*. The chromosome number of *C. indicum* (GD) was 2*n* = 36 (top panel) and that of *C. indicum* (HB) was 2*n* = 18 (bottom panel). Bar: 5 µm.

The diploid forms of *C. indicum* and *C. nankingense* are narrowly distributed and habitat specific (Yang et al., 2006; Li et al., 2014), whereas the tetraploid form of *C. indicum* is geographically widespread relative to its diploid ancestors. The following eight polyploid accessions were collected and planted in Guangzhou, China: three diploid forms of *C. indicum*, two diploid forms of *C. nankingense*, and three tetraploid forms of *C. indicum*. A global increase in flower size was observed in the tetraploid forms compared with their diploid counterparts (Figure 1A). The geographical distribution of the eight accessions is presented in Table 1 and Figure 1B.

**Table 1:** Plant material used, including the location, mean DNA content, and ploidy level of *Chrysanthemum*-related species

Species	Locality	Sample code	Latitude/°	Longitude/°	Genome size (G)	DNA content (2C, pg)	Ploidy level	Chromosome count	*N*
*C. indicum*	China: Huangshi, Hubei	Ci-HB_2__*x*_	30.22	115.08	3.22 ± 0.01	6.75 ± 0.01	2*x*	2*n *=* *18	3
	China: Anqi, Anhui	Ci-AH_2__*x*_	30.52	117.05	2.93 ± 0.03	6.13 ± 0.06	2*x*	2*n *=* *18^*^	3
	China: Jinzhong, Shanxi	Ci-SX_2__*x*_	37.70	112.74	2.66 ± 0.00	5.57 ± 0.00	2*x*	2*n *=* *18^*^	3
	China: Guangzhou, Guangdong	Ci-GD_4__*x*_	23.13	113.28	5.30 ± 0.03	11.10 ± 0.07	4*x*	2*n *=* *36	3
	China: Yichun, Jiangxi	Ci-JX_4__*x*_	27.80	114.39	6.00 ± 0.01	12.56 ± 0.02	4*x*	2*n *=* *36^*^	3
	China: Huanggang, Hubei	Ci-HB_4__*x*_	30.45	114.88	5.01 ± 0.02	10.50 ± 0.03	4*x*	2*n *=* *36^*^	3
*C. nankingense*	China: Nanjing, Jiangsu	Cn-JS_2__*x*_	32.04	118.77	2.77 ± 0.01	5.81 ± 0.02	2*x*	2*n *=* *18^*^	3
	China: Huanggang, Hubei	Cn-HB_2__*x*_	30.45	114.88	2.80 ± 0.04	5.86 ± 0.08	2*x*	2*n *=* *18^*^	3

*Notes*: The chromosome numbers of Ci-HB_2__*x*_ and Ci-GD_4__*x*_ (bold fonts) were counted from Figure 1C, whereas all others marked with an asterisk are inferred on the basis of the DNA content. Standard errors were calculated from three biological replicates for each assay.

The ploidy level was determined by counting the number of chromosomes and through flow cytometry. The DNA content (2C values) of the eight individuals sampled was divided into two groups, namely 5.57–6.75 pg and 10.50–12.56 pg, which appeared to correspond to two ploidy levels, (i.e. diploid and tetraploid, respectively; Table 1). Furthermore, we evaluated chromosomes in two representatives of *C. indicum* from Hubei and Guangdong Provinces. The ploidy levels of the eight sampled accessions of *Chrysanthemum* were inferred through flow cytometry (Figure 1C and Table 1). Generally, the accessions were grouped by the ploidy level.

### Cytotypes and genotypes affected specialized metabolism of FRs

**Figure 2:**
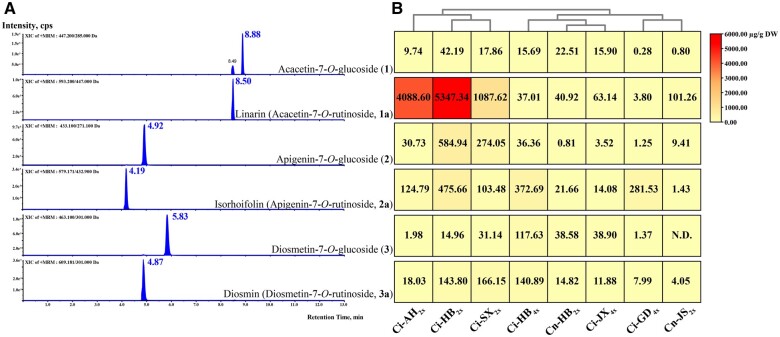
Major flavone glycosides accumulated in the flowers of different accessions of *Chrysanthemum* plants. A, Extracted-ion chromatogram of flavone glucosides and rutinosides standards. B, Heatmap visualization indicated the metabolic abundance of targeted FGs and FRs in different accessions of eight *Chrysanthemum* plants. The contents of major FG and FR compounds ranged from 0 to 6000 μg/g dry weight (DW). Linarin was highly accumulated in the *C. indicum* (HB_2__*x*_). Each datum is averaged from triplicates. N.D. indicates “not detected.”

Apart from morphological variations, the contents of targeted FGs (i.e. acacetin-7-*O*-glucoside, apigenin-7-*O*-glucoside, and diosmetin-7-*O*-glucoside) and FRs (i.e. acacetin-7-*O*-rutinoside, apigenin-7-*O*-rutinoside, and diosmetin-7-*O*-rutinoside) in flowers substantially differed among the eight accessions (Figure 2; Supplemental Figure S1 and Supplemental Table S1). The content of linarin, a dead-end metabolite, was higher in the diploids than in the tetraploids, especially in Hubei Province (Figure 2B). Moreover, isorhoifolin was highly accumulated in the Ci-HB of both the diploids and tetraploids (Figure 2B). The diosmin content differed within the identical cytotypes (different genotypes) and between different cytotypes (Figure 2B). These results suggest that cytotypes and genotypes affect specialized plant metabolites, resulting in an increase or decrease in their production.

### Screening and molecular cloning of *Chrysanthemum* 1,6RhaTs and phylogenetic analysis

On the basis of the findings of our previous transcriptomic analysis of tetraploid Ci-GD (Jiang et al., 2019) and the local Blast result obtained using *Citrus* 1,6RhaT, we examined five full-length cDNAs with >1,000 raw reads in the RNA-seq database (Supplemental Table S2) to determine the link between flavone glucosides and rutinosides. Among these genes, the deduced amino acid sequence of unigene0007998 was 51%–60% identical to the amino acid sequences of the 1,6RhaTs of flavanone, flavonol, and anthocyanidin (Supplemental Table S2; Kroon et al., 1994; Frydman et al., 2013; Rojas Rodas et al., 2014; Ohashi et al., 2016; Koja et al., 2018). The other four candidates exhibited <30% sequence similarity to known RhaTs. The cloning, recombinant protein expression induction, and purification of all the five candidate genes were performed. The preliminary biochemical assay indicated that only unigene0007998 catalyzed the formation of FRs (i.e. linarin, isorhoifolin, and diosmin) in vitro; thus, we named unigene0007998 as CiRhaT-GD_4x_.

To determine the spatial- and temporal-specific expression of CiRhaT-GD_4__*x*_, the total RNA was extracted from different tissues. Reverse transcription quantitative PCR (RT-qPCR) values of transcripts indicated that CiRhaT-GD_4__*x*_ was preferentially expressed in the flower bud (Supplemental Figure S2A). Furthermore, given that the conjugation of Rha (l-rhamnose) to specialized metabolites occurs in the cytoplasm (Jiang et al., 2021), its subcellular localization should be determined. The fluorescence of CiRhaT-GD_4__*x*_ infused with green fluorescent protein (GFP) was mainly observed in the cytosol (Supplemental Figure S2C).

By using the homology cloning strategy, we obtained the sequences of 1,6RhaTs in the other seven accessions. All of them contain a conserved motif, the plant secondary product glycosyltransferase (PSPG) box, which is involved in the binding of UDP-sugars. We noted 100% identity at the nucleotide level in two diploid *C. indicum* (i.e. CiRhaT-HB_2__*x*_ and CiRhaT-AH_2__*x*_; Table 2 and Supplemental Figure S3). Two tetraploid *C. indicum* (i.e. CiRhaT-HB_4__*x*_ and CiRhaT-GD_4__*x*_) exhibited 100% identity at the nucleotide level (Table 2 and Supplemental Figure S3). Thus, the six 1,6RhaTs were submitted to the UGT nomenclature committee and assigned the names UGT79A20, UGT79A21, UGT79A22, UGT79A23, UGT79A24, and UGT79A25, respectively (Table 2).

**Table 2:** List of *Chrysanthemum* CiRhaT-GD_4__*x*_ and their related RhaTs

Species	Locality	Gene product	UGT	Size (aa)^a^	Identity to CiRhaT-GD_4__*x*_ （%）	Origin
*C. indicum*	Guangzhou, Guangdong	CiRhaT-GD_4__*x*_	UGT79A20	469	100	Flower
*C. indicum*	Huanggang, Hubei	CiRhaT-HB_4__*x*_	UGT79A20	469	100	Flower
*C. indicum*	Yichun, Jiangxi	CiRhaT-JX_4__*x*_	UGT79A21	469	98.51	Flower
*C. indicum*	Huangshi, Hubei	CiRhaT-HB_2__*x*_	UGT79A22	469	97.65	Flower
*C. indicum*	Anqi, Anhui	CiRhaT-AH_2__*x*_	UGT79A22	469	97.65	Flower
*C. indicum*	Jinzhong, Shanxi	CiRhaT-SX_2__*x*_	UGT79A25	469	95.74	Flower
*C. nankingense*	Huanggang, Hubei	CnRhaT-HB_2__*x*_	UGT79A23	469	98.29	Flower
*C. nankingense*	Nanjing, Jiangsu	CnRhaT-JS_2__*x*_	UGT79A24	469	95.74	Flower

a aa, amino acids.

**Figure 3:**
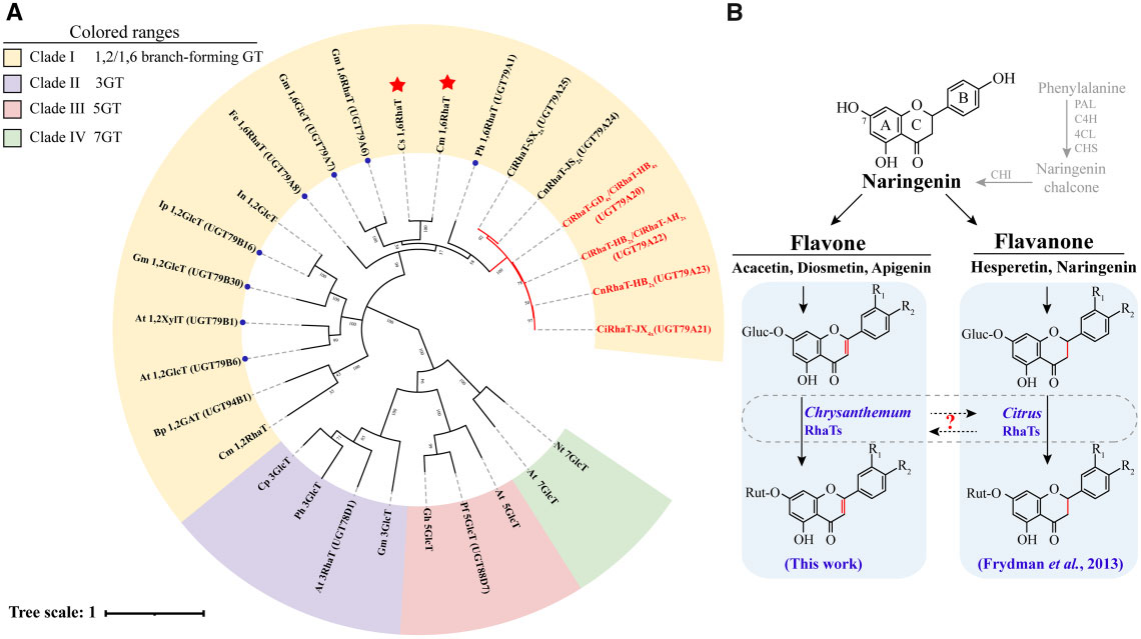
Molecular phylogenetic tree of *Chrysanthemum* RhaTs and other known branch-forming glycosyltransferases. A, Multiple sequences were aligned using Clustal W and used for tree construction with the maximum-likelihood method using MEGA7 and modified by iTOL. Bootstrap values (based on 1,000 replications) are indicated at each node. *Chrysanthemum* RhaTs are labeled with red subclades. The UGT79 family members are labeled with blue dots. Functional clade I of 1,2/1,6 branch-forming flavonoid UGTs is shaded yellow and detailed information is provided in Supplemental Table S3. Abbreviations and Genbank accession numbers of clades II–IV are as follows: *A. thaliana* 3RhaT (AEE31240), *Petunia hybrida* 3GlcT (AAD55985), *Citrus paradise* 3GlcT (GQ141630), *G. max* 3GlcT (P16166), *A. thaliana* 5Glc (AEE83370), *Perilla frutescens* 5GlcT (BAA36421), *Glandularia hybrida* 5GlcT (BAA36423), *Nicotiana tabacum* 7GlcT (AAB36653), and *A. thaliana* 7GlcT (AAL90934). GlcT, glucosyltransferase; XylT, xylosyltransferase; and GAT, galacturonic acid transferase. B, Schematic representation of the possible convergent evolution of RhaT proteins into 1,6-RhaTs in *Chrysanthemum* and *Citrus* plants. Enzymes labeled in the red star represent Cs1,6RhaT, flavanone-7-*O*-glucoside-1,6-RhaT from oranges (*C. sinensis*), Cm1,6RhaT, and flavanone-7-*O*-glucoside-1,6-RhaT from pummelo (*C. maxima*), whereas the putative RhaTs of *Chrysanthemum* are shown in the red subclade of (A). GT, glycosyltransferases; PAL, phenylalanine ammonia lyase; C4H, cinnamate 4-hydroxylase; 4CL, 4-coumaroyl-CoA ligase; CHS, chalcone synthase; and CHI, chalcone isomerase.

The phylogenetic tree constructed using the flavonoid glycosyltransferases revealed that UGTs for each specific flavonoid position (i.e. 3-O, 5-O, and 7-O) clustered together, whereas 1,2RhaTs and 1,6RhaTs belonged to a cluster of branch-forming UGTs (Figure 3A and Supplemental Table S3). In addition, six *Chrysanthemum* 1,6RhaTs formed a separate subclade with RhaTs that catalyze the rhamnosylation of flavonoid-7-*O*-glucoside and flavonoid-3-*O*-glucoside at the hydroxy group on C6 of the glucose moiety (Figure 3A). The 1,6RhaTs of *Chrysanthemum* and *Citrus* were divided into two subclades (Figure 3A). In *Citrus*, Cm1,6RhaT and Cs1,6RhaT are mainly involved in the conversion of flavanone glucosides to rutinosides (Frydman et al., 2013; Ohashi et al., 2016). The difference between flavanone and flavone is that flavanone lacks the double bond at the 2,3-position of the C-ring. By contrast, 1,6RhaTs in *Chrysanthemum* might catalyze the formation of the 1,6 sugar–sugar bond in flavone glucosides (Figure 3B).

### Four *Chrysanthemum* 1,6RhaTs are substrate-promiscuous enzymes catalyzing the branched-chain rhamnosylation of flavonoids glycosylated at position 7

Because the subclade of 1,6RhaTs differs between *Citrus* and *Chrysanthemum*, we determined whether Cm1,6RhaT and Cs1,6RhaT utilize FGs as substrates. Therefore, we performed gene synthesis following the Genebank ID (DQ119035 for Cm1,6RhaT and LC057678 for Cs1,6RhaT). The rhamnosylation activities of two *Citrus* and six *Chrysanthemum* 1,6RhaTs were investigated using *Escherichia coli*-expressed recombinant proteins. The recombinant proteins were purified using Dextrin Beads 6FF through maltose-binding protein (MBP) affinity chromatography for functional characterization (Supplemental Figure S4).

**Figure 4:**
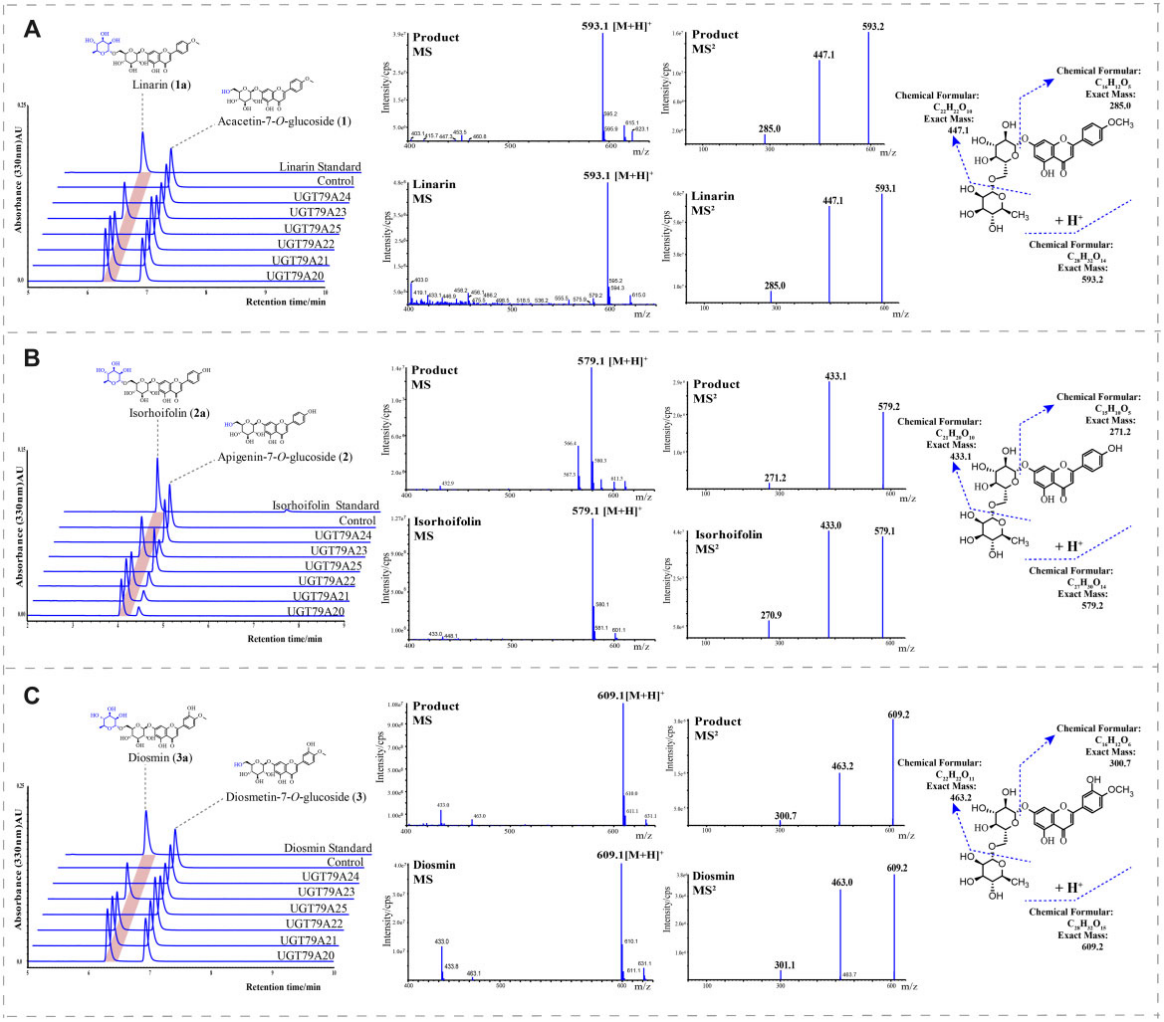
In vitro enzyme assays indicated that some RhaTs can catalyze FGs to the corresponding FRs (i.e. linarin, isorhoifolin, and diosmin). The reaction catalyzed by *Chrysanthemum* RhaTs involves the transfer of rhamnose from a sugar donor, UDP-Rha, to acceptor substrates. The MS/MS spectrum of the standard and products in the reaction of RhaTs. The selected acceptor substrates include acacetin-7-*O*-glucoside (A), apigenin-7-*O*-glucoside (B), and diosmetin-7-*O*-glucoside (C).

**Figure 5:**
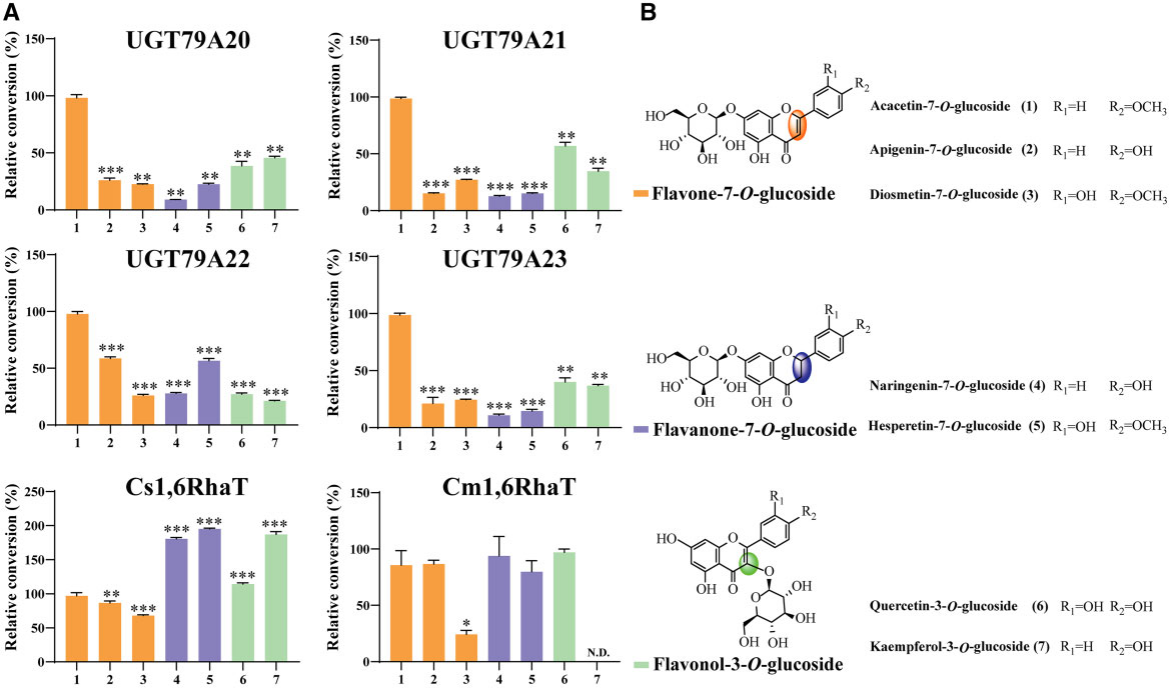
Sugar acceptor specificity of RhaTs of *Chrysanthemum* and *Citrus* plants. A, The relative percent conversions of rhamnosylated products catalyzed by RhaTs. The rhamnosylating activity toward acacetin-7-*O*-glucoside is considered to be 100%. Error bars represent the standard deviation from three replicates, *P <* *0.05, **P <* *0.01, and ***P <* *0.001 compared with acacetin-7-*O*-glucoside (**1**) by Dunnett’s multiple comparison test. N.D. indicates “not detected.” B, Chemical structures of substrates used as rhamnosyl acceptors.

Among the six *Chrysanthemum* 1,6RhaTs, four enzymes (UGT79A20, A21, A22, and A23) catalyzed acacetin-7-*O*-glucoside (**1**), apigenin-7-*O*-glucoside (**2**), and diosmetin-7-*O*-glucoside (**3**) to acacetin-7-O-rutinoside (**1a**), apigenin-7-*O*-rutinoside (**2a**), and diosmetin-7-*O*-rutinoside (**3a**), respectively (Figure 4). However, UGT79A24 (CnRhaT-JS_2__*x*_) and UGT79A25 (CiRhaT-SX_2__*x*_) from diploid *C. nankingense* and *C. indicum*, respectively, were nonfunctional, possibly because of amino acid mutations. Liquid chromatography followed by tandem mass spectrometry (LC-MS/MS) was performed to determine whether the product of **1a** exhibited a molecular ion at a *m*/*z* value of 593.1 [M-H]^+^, which is consistent with that of linarin (C_28_H_32_O_14_, 593.1). This *m*/*z* value increased by 146 (corresponding to the molecular weight of the rhamnose moiety) from a *m*/*z* value of 447.1 (corresponding to the molecular weight of acacetin-7-*O*-glucoside). The catalytic products of **2a** and **3a** were identified through LC-MS/MS through a comparison with their reference standards. We observed that Cm1,6RhaT and Cs1,6RhaT converted the aforementioned three flavone glucosides to the respective rutinosides (Figure 5), suggesting that 1,6RhaTs from *Citrus* and *Chrysanthemum* share a common evolutionary origin.

### Acacetin-7-*O*-glucoside is a preferred substrate of *Chrysanthemum* 1,6RhaTs

Except for the three FGs, hesperetin-7-*O*-glucoside (**4**), naringenin-7-O-glucoside (**5**), quercetin-3-*O*-glucoside (**6**), and kaempferol-3-*O*-glucoside (**7**) were rhamnosylated by four *Chrysanthemum* 1,6RhaTs (Figure 5). *Chrysanthemum* 1,6RhaTs catalyzed the branched-chain rhamnosylation of flavones, flavanones, and flavonols, catalyzing the rhamnosylation of both flavonoid-7-*O*-glucoside and 3-*O*-glucoside substrates at position 6 of the glucose moiety. Citrus fruits contain large quantities of flavanone glycosides (Frydman et al., 2013) and *Chrysanthemum* flowers contain abundant flavone glycosides (Shao et al., 2020; Yuan et al., 2020). The results of substrate specificity analysis indicated that all the four *Chrysanthemum* 1,6RhaTs exhibited a greater preference for acacetin-7-*O*-glucoside compared with other substrates, including flavanone and flavonol compounds (Figure 5A). By contrast, Cs1,6RhaT exhibited a greater preference for flavanones, with its relative activity being approximately two-fold that of flavones (Figure 5A).

**Figure 6:**
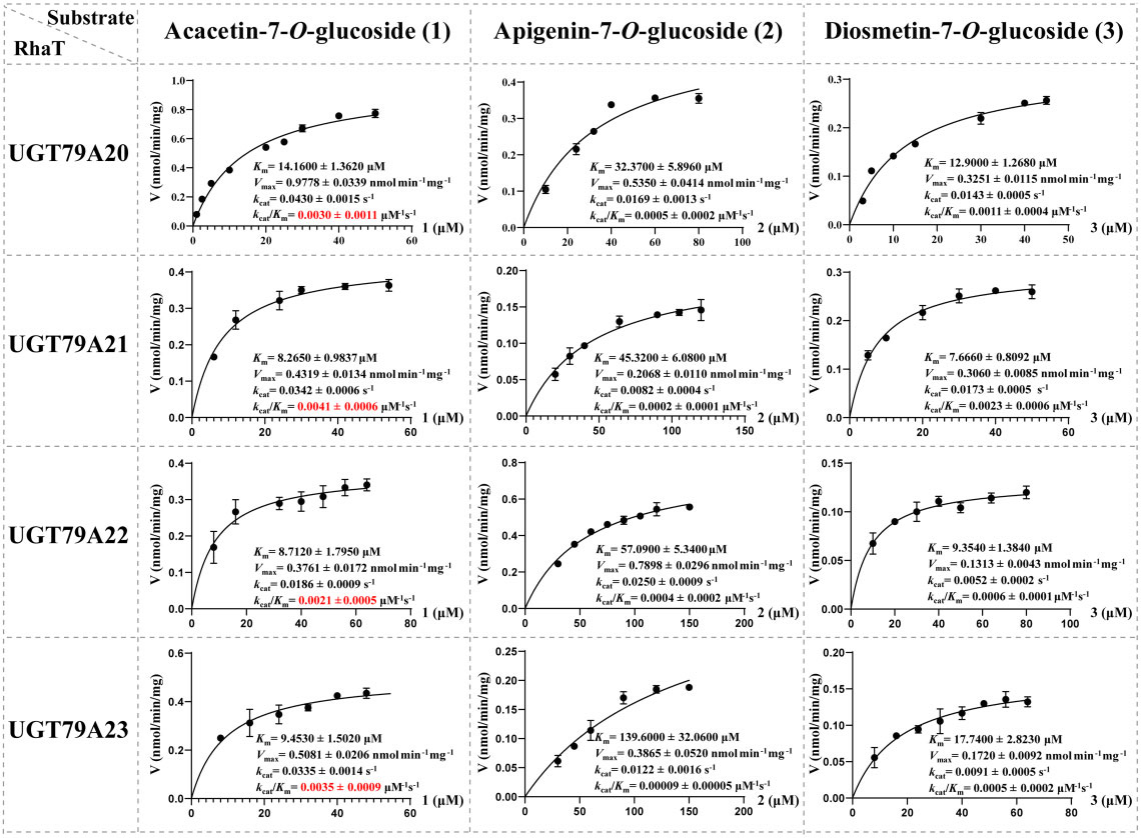
Kinetic analysis of *Chrysanthemum* RhaTs by using acacetin-7-*O*-glucoside (**1**), apigenin-7-*O*-glucoside (**2**), and diosmetin-7-*O*-glucoside (**3**) as acceptors. Data are means ± sd of three independent experiments.

To evaluate the catalytic efficiencies of *Chrysanthemum* 1,6RhaTs, we examined kinetic parameters by using compounds (**1**), (**2**), and (**3**) as substrates, which are naturally found in *Chrysanthemum* (Figure 6). The calibration curve of flavonoid rutinosides exhibited good linearity (*R*^2^ ≥ 0.999; Supplemental Figure S5). For each compound, the catalytic efficiency (*k*_cat_/*K*_m_) was similar among the four *Chrysanthemum* 1,6RhaTs (Figure 6); this finding is in line with the fact that in many cases, gene duplication events yield multiple copies of isozymes without changes in the catalytic function (Weng, 2014). By contrast, all the four *Chrysanthemum* 1,6RhaTs exhibited higher substrate specificity for acacetin-7-*O*-glucoside than did the other two compounds (Figure 6); this result is consistent with the higher accumulation of linarin than that of the other two rutinosides in the *Chrysanthemum* diploid ancestor (Figure 2). These findings indicate that *Chrysanthemum* 1,6RhaTs are mainly responsible for the biosynthesis of linarin (**1a**) and exhibit the highest relative activity and catalytic efficiency for compound **1**.

### *Chrysanthemum* 1,6RhaT contributes to the biosynthesis of FRs in planta

**Figure 7:**
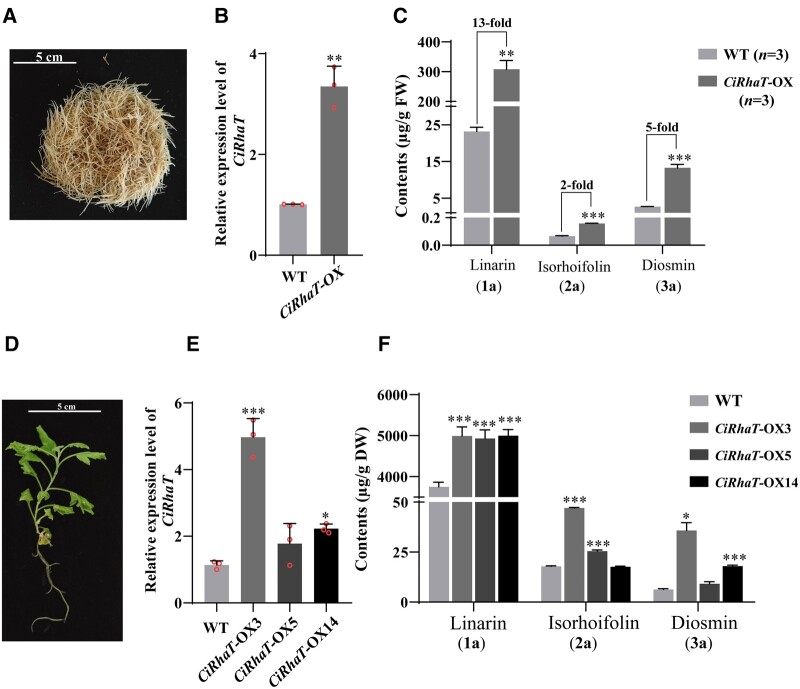
In vivo functions of CiRhaT-GD_4__*x*_ enzymes in *C. indicum*. A–C, Overexpression of *CiRhaT-GD_4x_* in hairy root cultures by *A. rhizogenes*. A, Hairy root cultures of *C. indicum*. Scale bar: 5 cm. B, Expression level of *CiRhaT* in transgenic hairy roots. C, Effect of the overexpression of *CiRhaT* on the biosynthesis of FRs. Data are presented as the mean ± sd (*n *=* *3 biologically independent samples). *P <* *0.05, **P <* *0.01, and ***P <* *0.001 compared with the WT group by using Student’s *t* test. D–F, Overexpression of *CiRhaT-GD_4x_* in *Chrysanthemum* plant by *Agrobacterium tumefaciens*. D, The phenotype of the transgenic line. E, Comparative expression analysis of *RhaT* in transgenic and WT plants through RT-qPCR. F, Flavonoid rutinoside contents in the transgenic lines. For (E) and (F), experiment was performed in triplicate and error bars represent standard deviation, asterisks indicate a significant difference from WT control line (*P <* *0.05, ***P <* *0.001) analyzed through one-way ANOVA with Dunnett’s multiple comparison test.

To investigate the functions of RhaT enzymes in planta, we cloned the full-length cDNA of CiRhaT-GD_4__*x*_ (UGT79A20) into pCAMBIA-1302 under the control of a CaMV 35S promoter. Subsequently, the construct was transformed into diploid *C. indicum* by using *Agrobacterium rhizogenes* to obtain transgenic hairy roots (Figure 7A and Supplemental Figure S6, A–C). The hairy roots induced by K599 were considered as the wild type (WT). The presence of transgenes was confirmed through genomic PCR (Supplemental Figure S6 D). In the hairy root tissue cultures of the *CiRhaT*-overexpressing line, *CiRhaT* exhibited a 3.3-fold higher expression than did the control group (Figure 7B). Moreover, the contents of the FRs **1a**, **2a**, and **3a** increased significantly by 13-, 2-, and 5-fold respectively, compared with the WT group (Figure 7C).

We evaluated *CiRhaT* overexpression in *C. indicum* by using an *Agrobacterium tumefaciens*-mediated method, generating three independent transgenic lines, which were verified through genomic PCR (Figure 7D and Supplemental Figure S7). RT-qPCR results revealed that transcripts levels were substantially increased in the transgenic lines, exhibiting 4.4-, 1.6-, and 2-fold increases in *CiRhaT*-OX3, *CiRhaT*-OX5, and *CiRhaT*-OX14 compared with the WT plant (Figure 7E). After a growth period of 75–90 days, the contents of three FRs (**1a**, **2a**, and **3a**) increased to 1.3-, 1.7-, and 3.3-fold of that in the WT plant, respectively (Figure 7F). These results indicate that 1,6RhaT is involved in the biosynthesis of FRs in *C. indicum* and functions as 1,6-RhaT.

## Discussion

### Molecular evolution of 1,6RhaT in *Chrysanthemum* polyploids and its contribution to FR biosynthesis

The *Chrysanthemum* genus is closely associated with hybridization and polyploidization, with *Chrysanthemum* species exhibiting diverse ploidy levels (Wang et al., 2014; Won et al., 2017). The species and cytotypes of *C. indicum_2x_*, *C. nankingense_2x_*, and *C. indicum_4x_* are suitable for studying not only morphology but also specialized metabolism in polyploidy evolution (Figures 1 and 2).

The differences in morphological characteristics and 1,6RhaT sequences between *C. indicum_2x_* and *C. indicum_4x_* in this study may have originated through allopolyploidization. Hybridization and polyploidization are prevalent in the evolution of the genus *Chrysanthemum*. In this study, we observed white petals in Ci-HB_4__*x*_ (Figure 1). High carotenoid cleavage dioxygenase (CCD4a) expression contributes to white color formation in *C. morifolium_6x_* petals (Ohmiya et al., 2006). Thus, tetraploid *C. indicum* with white color petals (observed in this study) might be a parental species of the white flower phenotype in *C. morifolium*. The 100% nucleotide sequence identity between *CiRhaT-HB_4x_* and *CiRhaT-GD_4x_* is in agreement with similar metabolites levels observed between Ci-HB_4__*x*_ and Ci-GD_4__*x*_ (Table 2 and Figure 2), indicating that tetraploid Ci-HB and Ci-GD share a common origin. Thus, CCD4 was observed to be highly expressed in the flower petals of Ci-HB_4__*x*_.

A 100% nucleotide sequence identity was noted between *CiRhaT-HB_2x_* and *CiRhaT-AH_2x_* (Table 2), indicating that the cytotypes of diploid Ci-HB and Ci-AH may share a common origin. This finding is in accordance with that of the phylogenetic analysis of flavone glycosides, where Ci-HB_2__*x*_ and Ci-AH_2__*x*_ fell into the same clade (Figure 2B). The 2*x* and 4*x* cytotypes of *C. indicum* are distributed in the Shen-Nong-Jia Mountain of Hubei (HB) Province, China, and are ecologically differentiated. The diploids are strictly limited to the summit area, whereas the tetraploids are widely distributed in the medium- and low-altitude regions of Shen-Nong-Jia Mountain (Yang et al., 2006). The high accumulation of linarin in Ci-HB_2__*x*_ might be associated with the high altitude. Similarly, the high accumulation of maysin and rhamnosyl isoorientin was observed in the leaves of the high-altitude landraces of maize after UV-B exposure (Casati and Walbot, 2005). Furthermore, a negative association between the lower latitude and flavonoid content has been demonstrated in Common Juniper leaves (Martz et al., 2009) and *Ruellia* (Tripp et al., 2018). Similarly, our finding of a lower flavone content (e.g. linarin) in Ci-GD_4__*x*_ might be associated with the lower latitude (Table 1 and Figure 2). Polyploidy enhances secondary metabolite production in plants (Madani et al., 2021); however, a recent comparative metabolomic analysis of doubled diploids and their diploid citrus rootstock (*Citrus junos* cv. Ziyang xiangcheng) provided conflicting information on the effects of polyploidization on metabolites. The 33 identified flavones were downregulated in tetraploid *Citrus* species compared with in diploid species (Tan et al., 2015). The contents of three FRs in *C. nankingense_2x_* were substantially lower than those in the closely related species *C. indicum_2x_* (Figure 2B), suggesting that they evolved independently. This speculation is supported by the findings of a previous study of the molecular markers of the *Chrysanthemum* polyploid complex (Yang et al., 2006).

CnRhaT-JS_2__*x*_ and CiRhaT-SX_2__*x*_ (UGT79A24 and UGT79A25, respectively) exhibited more nonsynonymous substitutions than did UGT79A20, A21, A22, and A23 (Supplemental Table S4 and Supplemental Figure S8). These two 1,6RhaTs lost the catalytic function possibly due to accumulating deleterious mutants. Four copies were noted in the genome of Ci-HB_2__*x*_ (unpublished data). The genome of Cn-JS_2__*x*_ and Ci-SX_2__*x*_ might contain more than one copy of 1,6RhaT. Even if one copy becomes a full-length nonfunctional gene, the other remains functional. This could explain the accumulation of FRs observed in Cn-JS_2__*x*_ and Ci-SX_2__*x*_ (Figure 2). Functional and nonfunctional RhaTs occurred in the diploid progenitor species (Figure 4). A duplicate of all genes is present in the genome immediately following a whole-genome duplication event. In *Chrysanthemum* allopolyploidization, duplicated gene pairs can undergo gene losses or frame shifts, which occurred in the glutathione *S*-transferase (GST) gene family of soybean, where 72% of duplicated GST gene pairs experienced gene losses or pseudogenization (Liu et al., 2015). This can be a reason for a substantial decrease in linarin accumulation in tetraploid *Chrysanthemum* plants.

**Figure 8:**
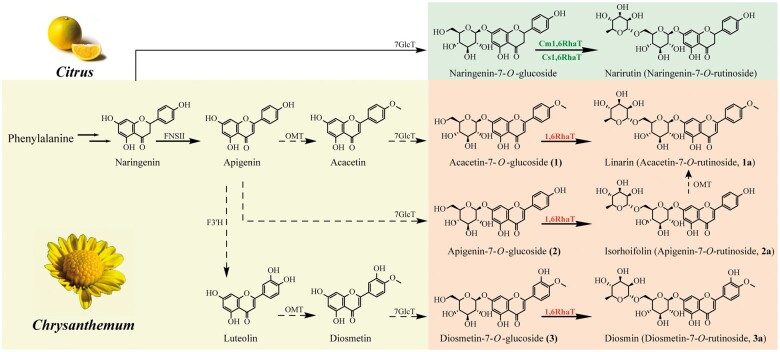
Biosynthetic pathway of proposed FRs in *Chrysanthemum* and specialized flavonone rutinosides in *Citrus*. Genes encoding OMT, 7GlcT, and F3ʹH were presumably involved in pharmaceutically active linarin, isorhoifolin, and diosmin biosynthesis. Dashed arrows indicate proposed steps. Enzymes highlighted in red represents 1,6RhaT that has been biochemically characterized in this study. FNS II, flavonoid synthase II; F3ʹH, flavonoid-3ʹ-hydroxylase; 1,6RhaT, flavonoid-7-*O*-glucoside-1,6-RhaT; Cm1,6RhaT, flavanone-7-*O*-glucoside-1,6-RhaT from pummelo (*Citrus maxima*); Cs1,6RhaT, flavanone-7-*O*-glucoside-1,6-RhaT from oranges (*Citrus sinensis*).

For the same FG substrate, the enzyme kinetic parameters of four 1,6RhaTs (UGT79A20–A23) were similar (Figure 6), suggesting that the catalytic functions of *C. indicum_2x_*, *C. nankingense_2x_*, and *C. indicum_4x_* were unchanged. The profile variation between cytotypes and accessions might not be determined by examining dynamic catalytic activity. However, the metabolite profile and content can be evaluated on the basis of gene expression levels. For example, *RhaT* exhibited a 2.4-fold higher expression in Ci-HB_2__*x*_ than in Ci-GD_4__*x*_ (Supplemental Figure S2B). Apart from 1,6RhaT, *O*-methyltransferase (OMT), flavonoid-3-hydroxylase (F3′H), and 7GlcT could be involved in FR biosynthesis (Figure 8). Thus, the concerted gene expression, distinct spatiotemporal expression patterns, transcription factors, and transcription regulatory network might contribute to differences in the FR content and metabolic diversification.

### Convergent molecular evolution of genes encoding 1,6RhaT in *Chrysanthemum* and *Citrus*

Although genes encoding 1,6RhaT from pummelo (*Citrus maxima*) and orange (*C. sinensis*) have been functionally characterized, their biochemical characterization by using acacetin-7-*O*-glucoside and apigenin-7-*O*-glucoside as substrates is yet to be performed (Frydman et al., 2013; Ohashi et al., 2016). In this study, genes encoding Cm1,6RhaT and Cs1,6RhaT were recloned by performing gene synthesis. The biochemical results revealed that two *Citrus* 1,6RhaTs catalyzed the specialized metabolites of *Chrysanthemum* (i.e. flavone glucosides **1**, **2**, and **3**) and four *Chrysanthemum* 1,6RhaTs catalyzed the rhamnosylation of abundant flavonoid skeletons (e.g. flavanone glucosides) in *Citrus* (Figure 5), suggesting that they share a common evolutionary origin.

The substrate preference of *Citrus* 1,6RhaT was not determined due to the lack of availability of UDP-Rha. In this study, the substrate preference of 1,6RhaT was compared between *Chrysanthemum* and *Citrus* because of the availability of commercial UDP-Rha. The results of the biochemical assay revealed that *Citrus* 1,6RhaTs not only used flavanone glucosides but also flavone glucosides to produce the respective FRs in vitro (Figure 5), although flavone glucosides might be rarely present in *Citrus* plants. Notably, the phylogenetic analysis of RhaTs from *Citrus* and *Chrysanthemum* families was both located in the same clade together with RhaTs from other families in the tree, suggesting that their common ancestor possessed rhamnosylation activity (Figure 3A). The 1,6RhaT putative orthologs in *Chrysanthemum* and *Citrus* catalyzed the same reactions, indicating that the convergent molecular evolution of genes encoding 1,6RhaT might occur in *Chrysanthemum* and *Citrus*, thus increasing our understanding of the roles of flavonoid rutinosides in their biosynthetic pathways.

### RhaT mainly contributes to linarin biosynthesis in *Chrysanthemum* plants

RhaT enzymes caused considerable biochemical complexity in vitro due to their ability to rhamnosylate multiple substrates in a pathway (Figure 5). Two biosynthetic pathways can contribute to linarin biosynthesis (Figure 8). In the first pathway, apigenin might be sequentially *O*-methylated, glucosylated, and rhamnosylated to generate linarin. In the second pathway, glucosylation and rhamnosylation can result in the generation of isorhoifolin, followed by *O*-methylation, to produce linarin (Figure 8). Acacetin-7-*O*-glucoside (**1**) was the preferred substrate for RhaT in vitro (Figures 5 and 6). Thus, we used RNA interference (RNAi) methods to confirm the in vivo functions of RhaT in *Chrysanthemum*. However, the plant did not survive after the RNAi technique (Supplemental Figure S9). Thus, we used RhaT overexpression in the hairy roots to determine RhaT function in planta. RhaT overexpression led to a higher content of linarin than those of isorhoifolin and diosmin (Figure 7C). Moreover, the biochemical experiment in our study revealed that OMT cannot catalyze isorhoifolin to generate linarin (unpublished data). These results indicate that RhaT mainly contributes to linarin biosynthesis in *Chrysanthemum* plants.

### An alternative strategy for the future synthesis of pharmaceutically active diosmin

In addition to flavanones, which are the major flavonoids found in citrus fruits, species of the *Citrus* genus produce a small amount of flavones (e.g., diosmin) (Marin and Del Rio, 2001; Caristi et al., 2003; Dugo et al., 2005; Saeidi et al., 2011; Barberis et al., 2020; Klimek-Szczykutowicz et al., 2020). Moreover, isorhoifolin has been found only in *Citrus* extracts (e.g. fruit juices) (Kanaze et al., 2003; Ramful et al., 2010; Ballester and Lafuente, 2017). The specialized metabolic pathways of diosmin and isorhoifolin remain to be elucidated. Currently, diosmin is obtained through the dehydrogenation of the flavanone glycoside hesperidin. Hesperidin is abundantly found in the pericarp of several citrus fruits and in some medicinal herbs, from which it is extracted and then converted to diosmin (Gerges et al., 2022). *Chrysanthemum* 1,6RhaT can be an alternative to hemisynthesis for pharmaceutically active diosmin and isorhoifolin through the synthetic biology platform.

## Conclusions

Taken together, *Chrysanthemum* accumulates abundant flavone glycosides, which provide excellent health benefits. In this study, we first collected and inferred the ploidy level of the eight *Chrysanthemum* accessions with diverse flavone glycoside profiles. Next, we isolated genes encoding the RhaTs of FRs from the *Chrysanthemum* accessions and performed their functional characterization in vitro and in vivo. Furthermore, *Chrysanthemum* RhaTs were biochemically and phylogenetically compared with *Citrus* 1,6RhaTs to obtain molecular evolutionary insights into 1,6RhaTs in plants. Our findings are valuable for studies on medicinal and ornamental species and elucidating specialized metabolic pathways.

## Materials and methods

### Plant material and chemical compounds

Eight accessions of *Chrysanthemum* plants endemic to China are presented in Figure 1. All the samples were collected and transplanted to a greenhouse at Guangzhou University of Chinese Medicine (Guangdong, China).

Compounds **1**, **1a**, **2**, **2a**, **3**, **3a**, **4**, **4a**, **5**, **5a**, **6**, **6a**, **7**, and **7a** and UDP-rhamnose were purchased from Shanghai Yuanye Bio-Technology Co., Ltd, China.

### Flow cytometry measurement of nuclear DNA content

Fresh leaves were used for the measurement of the nuclear DNA content. The nuclear DNA content was determined using previously published FCM protocols (Dolezel et al., 2007). Tomato (*Solanum lycopersicum* L., 2C = 1.84 pg) was selected as the internal reference standard. The suspension of samples and reference was mixed at an appropriate proportion and then quantified using a flow cytometer (BD FACScalibur, USA) with 488-nm blue-light excitation. Three replicates were used for genome size estimations for each sample.

### Chromosome analysis

Vigorous root tips (1–3 cm in length) were excised from the seedlings and pretreated with saturated *p*-dichlorobenzene solution under darkness for 3.5 h. Subsequently, the roots were fixed with Carnoy solution for 2 h, rinsed with distilled water for 5 min, hydrolyzed with 1 mol/L HCl at 45°C for 45 min, rinsed with distilled water for 10 min, and stained with carbol fuchsin for 2 h. The microscopic examination was performed using a Nikon 80i microscope (Nikon, Japan).

### Extraction and profiling of FGs and FRs

The frozen flower samples (25 mg) were weighed and extracted at 4°C by using 0.6 mL of 70% (v/v) aqueous methanol, vortexed once every 30 min, for six times. Subsequently, the samples were centrifuged at 12,000 rpm for 10 min and diluted 15 times before LC-MS/MS analysis.

The sample extracts were analyzed using an LC-electrospray ionization (ESI)-MS/MS system (HPLC, Shim-pack UFLC SHI-MADZU CBM30A system; MS, Applied Biosystems 4500 QTRAP). For separation, a Waters Acquity UPLC HSS T3 C18 (2.1 × 100 mm, 1.8 µm) was used. The mobile phase consisted of 0.1% (v/v) formic acid in water (solvent A) and 0.1% (v/v) formic acid in acetonitrile (solvent B), with the flow rate set at 0.4 mL/min. Separation was achieved using a gradient starting at 5% B; increasing to 22% B in 1 min, held for 5 min; increasing to 40% B in 2 min; increasing to 95% B in 3 min, held for 1 min; and switching back to 5% B in 0.1 min, held for 2.9 min.

Linear ion trap and triple quadrupole scans were acquired on a triple quadrupole-linear ion trap mass spectrometer (API 4500 QTRAP LC-MS/MS system) equipped with an ESI mode Turbo Ion-Spray interface, operated in a positive ion mode, and controlled using Analyst 1.6 software (AB Sciex). A specific set of MRM transitions was monitored for each period according to metabolites eluted within this period.

### Screening and molecular cloning of *Chrysanthemum* 1,6RhaTs

The transcriptome derived from the five tissues of *C. indicum* L*.* (Guangdong Province, China) was used for searching RhaTs as reported previously (Jiang et al., 2019). Then, a local BLASTP analysis for candidate RhaT sequences using the sequence of *Citrus* 1,6RhaT was performed, and five unigenes exhibiting high identity were chosen as RhaT candidates in this study. Then, other *Chrysanthemum* 1,6RhaT fragments were selected on the basis of their homology with *CiRhaT1-GD_4x_* and amplified from the cDNA of seven *Chrysanthemum* accessions by using gene-specific primers for CiRhaT1-GD_4__*x*_-F and CiRhaT1-GD_4__*x*_-R (Supplemental Table S5).

The candidate RhaTs were cloned into the pMAL-c5X vector by using the homologous recombination method at the *Nde*I and *Hind*III site and verified through sequencing. The plasmids of pMAL-c5X-Cs1,6RhaT and pMAL-c5X-Cm1,6RhaT were synthesized by a company (BGI-Write, China).

### Heterologous expression of *Chrysanthemum* 1,6RhaTs protein

The recombinant vectors were transformed into *E. coli* Rosetta (DE3) cells for heterologous expression. The recombinant *E. coli* cells were harvested through centrifugation and resuspended in buffer A (20 mM Tris–HCl, 200 mM NaCl, 1 mM EDTA, and 1 mM DTT, pH 7.4). The lysate was centrifuged to remove insoluble cell debris. The supernatant was applied to a Dextrin Bead 6FF column (25 mL, Smart Life Sciences, China) that was equilibrated with buffer A. The column was washed with buffer A containing 10 mM maltose, and 1-mL fractions were collected.

The active column-bound fractions were concentrated and desalted using PD-10 columns (GE Healthcare), followed by elution with buffer B (50 mM sodium phosphate monobasic buffer, pH 7.5). Sodium dodecyl sulfate-polyacrylamide gel electrophoresis was performed according to the method described by Laemmli (1970), and proteins in the gels were visualized using Coomassie Brilliant Blue R-250.

### Enzyme assays and kinetics

The standard reaction mixture (500 µL) consisted of 50 mM sodium phosphate monobasic (pH 7.5), 20 µM flavonoid glycoside substrate (compounds **1–7**), 100 µM UDP-β-l-rhamnose, and 20 µg purified RhaT. For negative control, enzymes boiled at 95°C for 10 min were used in place of active enzymes. After incubation at 37°C for 60 min, the reaction was stopped by the addition of 500 µL of methanol. The substrates and rhamnosylated products were analyzed using the Waters UPLC Acquity system at a flow rate of 0.3 mL/min at 40°C with an Acquity UPLC BEH C18 column (2.1 × 100 mm, 1.7 μm, Waters, USA). The mobile phase consisted of acetonitrile (solvent A) and H_2_O (0.1% formic acid, solvent B, v/v). A linear gradient was set as follows: 0–5 min, 85%–69% B; 5–12 min, 69%–62% B; 12–15 min, 62%–40% B; 15–16 min, 40%–85% B; and 15–16 min, re-equilibrated to the initial condition. Flavone glycosides and their rutinosides were detected by measuring absorbance at 330 nm. Flavanone glycosides and flavonol glycosides were detected at 280 and 260 nm, respectively. At least three independent reactions were performed for each substrate.

To determine kinetic parameters, enzyme assays were conducted using 5–150 µM acceptor substrates and 0.1 mM donor substrates. All experiments were performed in triplicate. Next, kinetic parameters were determined by performing a nonlinear regression analysis by using GraphPad Prism 6 software. After entering data, the analyze option, nonlinear regression, the panel of enzyme kinetics equations, and Michaelis–Menten equation were chosen in sequence to plot a substrate–velocity curve to determine *V*_max_ and *K*_m_.

### Subcellular localization

The encoding sequences of CiRhaT-GD_4__*x*_ were subcloned into the pCG3301-GFP vector by using *Sac*I and *Sal*I restriction sites (primers are in Supplemental Table S5). The plasmid was then introduced into *A. tumefaciens* strain GV3101. The empty vector was used as a control. The constructs were transiently transformed into *Nicotiana benthamiana* leaves for performing the localization assay. GFP fluorescence was observed using an Olympus FV3000 confocal microscope. Detection parameters were as follows: GFP: excitation 488 nm, emission 510 nm, laser intensity 2.9, gain 1, chloroplast autofluorescence: excitation 561 nm, emission 618 nm, laser intensity 1.0, and gain 1.

### Overexpression experiments in *C. indicum*

For overexpression experiments, the full-length DNA regions of *CiRhaT-GD_4x_* were amplified using primers listed in Supplemental Table S5. PCR products were inserted into the overexpression vector pCAMBIA-1302 with *Nco*I and *Spe*I restriction sites.

For hairy root-mediated overexpression, 4–6-week-old *C. indicum* (Ci-HB_2__*x*_) seedlings were stabbed using a sterile needle with the bacterial strain *A. rhizogenes* K599 containing pCAMBIA-1302-CiRhaT-GD_4__*x*_. Then, the hairy roots were transferred to MS medium supplemented with 500 mg/L of cefotaxime to prevent Agrobacterium overgrowth. After 1 month, the hairy roots were transferred to a liquid medium. The transgenic hairy roots were harvested for RNA isolation and metabolite extraction.

For *A. tumefaciens*-mediated overexpression, cotyledons and the first true leaves of the 30-day-old seedlings of *C. indicum* (Ci-HB_2__*x*_) were transformed. After *Agrobacterium*-mediated transformation, stem explants were cut into small fragments with a size of <3 cm, soaked in an *Agrobacterium* solution for 7 min, and cultured on a co-culture medium (MS solid medium containing 100 mM AS, pH 5.8) at 24°C ± 2°C under dark conditions for 48 h. After washing with sterile water, we transferred the explants into the bud-inducing medium (MS solid medium containing 1.5 mg/L 6-BA, 0.05 mg/L NAA, 500 mg/L Cefo, and 2.5 mg/L Hyg, pH 5.8). The medium was replaced every 2 weeks until green shoots grew to 2–3 cm. Then, the shoots were transferred to a rooting medium (0.5 MS solid medium containing 0.03 mg/L NAA, 40 mg/L Myo-inositol, 250 mg/L Cefo, and 5mg/L Hyg, pH 5.8). Hygromycin-resistant primary transformants were screened through genomic PCR. The flavonoid glycoside components in the leaves of the transgenic plants were analyzed through LC-MS/MS as described.

### RT-qPCR

To determine the transcript abundance of *CiRhaT-GD_4x_* in different tissues, the roots, leaves, stems, roots, flowers, and flower buds were collected from *C. indicum* (Ci-GD_4__*x*_). *CiRhaT-GD_4x_* fragments were amplified using RT-qPCR-CiRhaT-GD_4__*x*_ pairs (Supplemental Table S5). *EF1α* (KF305681) was used as the reference gene for normalization (Gu et al., 2011).

To determine the relative expression level of *RhaTs* at different ploidy levels, the flower buds of Ci-HB_2__*x*_ and Ci-GD_4__*x*_ were collected. All reactions were performed in triplicate and the *Chrysanthemum GAPDH* (KC508619) gene was used as the internal control.

RT-qPCR primers (Supplemental Table S5) were used to analyze the expression level of *CiRhaT* in the transgenic and WT plants by using leaf tissues or hairy roots. The 210-bp *Chrysanthemum EF1α* gene was used as the reference for normalization (Gu et al., 2011). Each reaction was performed using three biological replicates and verified by performing a melting curve analysis. The relative amounts of target genes were evaluated based on the relative expression index of mRNA by using the 2^−ΔΔC(T)^ method (Schmittgen and Livak, 2008).

### Statistical analyses

Unless specifically described, all the experiments in this article were repeated three times. GraphPad Prism (version 8.0) and SPSS (version 26.0) was used for the statistical analysis. Data are presented as means ± SD. The statistical evaluations used unpaired *t* tests and one-way analysis of variance (ANOVA) with multiple comparisons. The results were considered statistically significant at *P <* *0.05.

### Phylogenetic analyses

The sequences for phylogenetic analysis were obtained from National Center for Biotechnology Information (NCBI) database. The phylogenetic tree was constructed using MEGA 7.0 (Kumar et al., 2016) with the maximum-likelihood method based on Clustal W multiple alignments, with the JTT model. The phylogenetic analysis was colored for visualization by iTOL (Letunic and Bork, 2021). The bootstrap confidence values were obtained based on 1,000 replicates.

## Accession numbers

The gene sequences of *Chrysanthemum* 1,6RhaTs are deposited in GeneBank under the following accession numbers: CiRhaT-GD_4__*x*_/CiRhaT-HB_4__*x*_ (UGT79A20), OL422134; CiRhaT-JX_4__*x*_ (UGT79A21), OL422135; CiRhaT-AH_2__*x*_/CiRhaT-HB_2__*x*_ (UGT79A22), OL422136; CnRhaT-HB_2__*x*_ (UGT79A23), OL422137; CnRhaT-JS_2__*x*_ (UGT79A24), OL422138; and CiRhaT-SX_2__*x*_ (UGT79A25), OL422139.

## Supplemental data

The following materials are available in the online version of this article.

**Supplemental Figure S1.** Extracted-ion chromatogram (XIC) of the methanol extracts of *Chrysanthemum* plants.

**Supplemental Figure S2.** RT-qPCR analysis of *RhaT* transcripts in different tissues and organs and subcellular localization.

**Supplemental Figure S3.** Amino acid alignment of *Chrysanthemum* and *Citrus* RhaTs. *Dashes* represent gaps introduced to improve the alignment.

**Supplemental Figure S4.** Expression of recombinant RhaT enzymes in *E. coli*.

**Supplemental Figure S5.** Standard curves of seven flavonoid rutinoside compounds in determination of relative activity.

**Supplemental Figure S6.** Regeneration and culture of *C. indicum* hairy roots after infection with *A. rhizogenes*.

**Supplemental Figure S7.** Construction of the transformation system of *C. indicum.*

**Supplemental Figure S8.** Nucleotides and deduced amino acid sequence alignment of CiRhaT-SX_2__*x*_ and CiRhaT-JS_2__*x*_.

**Supplemental Figure S9.** Silencing effects of *CiRhaT-GD_4x_* gene in transgenic *Chrysanthemum*.

**Supplemental Table S1.** Parameters of linear regression and experimental retention times (*t*_R_), LOD, LOQ, and RSD (%) for the studied compounds through LC-MS/MS.

**Supplemental Table S2.** Amino acid sequences of CiRhaT-GD_4__*x*_ and some known 1,6-RhaTs were aligned and analyzed using Clustal W.

**Supplemental Table S3.** List of 1,6/1,2 glycosidic bond flavonoid UGTs used in the phylogenetic analysis.

**Supplemental Table S4.** Nonsynonymous substitution of RhaT sequences in *Chrysanthemum*.

**Supplemental Table S5.** For clone, vector and RT-qPCR primers used in this study. Restriction enzyme sites are highlighted by bold and underline format.

## Supplementary Material

kiac371_Supplementary_Data

## Notes

These authors contributed equally (Q-W.W., M.W., and L-F.F.)

D.-M.M. planned and designed the research. Q.-W.W., M.W., and L.-F.F. performed the experiments. M.W. collected the materials. Q.-W.W., L.D., W.-K.W., X.-J.L., and H.-L.L. analyzed the data. R.-T.Z. and J.-F.Y. coordinated the research project. D.-M.M. and Q.W.-W. discussed and revised the final version. All authors read and approved the final manuscript.

The author responsible for distribution of materials integral to the findings presented in this article in accordance with the policy described in the Instructions for Authors (https://academic.oup.com/plphys/pages/general-instructions) is: Dong-Ming Ma (madm@gzucm.edu.cn).
