# Supplementary material for: Rhamnosyltransferases involved in the biosynthesis of flavone rutinosides in Chrysanthemum species
Source: Plant Physiol. 2022 Aug 10;190(4):2122–36. doi: 10.1093/plphys/kiac371 (PMC9706480; doi:10.1093/plphys/kiac371)
Supplement: kiac371_Supplementary_Data [file kiac371_supplementary_data.pdf]

Supporting Information

Article title: Rhamnosyltransferases Involved in the Biosynthesis of Flavone Rutinosides in *Chrysanthemum* Species

Authors: Qing-Wen Wu, Min Wei, Ling-Fang Feng, Li Ding, Wu-Ke Wei, Jin-Fen Yang, Xiao-Jing Lin, Hui-Lin Liang, Ruo-Ting Zhan, and Dong-Ming Ma

The following Supporting Information is available for this article:

Supplemental Figure S1 Extracted-ion chromatogram (XIC) of the methanol extracts of *Chrysanthemum* plants.

Supplemental Figure S2 RT-qPCR analysis of *RhaT* transcripts in different tissues and organs and subcellular localization.

Supplemental Figure S3 Amino acid alignment of *Chrysanthemum* and *Citrus* RhaTs.

Supplemental Figure S4 Expression of recombinant RhaT enzymes in *E. coli*.

Supplemental Figure S5 Standard curves of seven flavonoid rutinoside compounds in determination of relative activity.

Supplemental Figure S6 Regeneration and culture of *C. indicum* hairy roots after infection with *A. rhizogenes*.

Supplemental Figure S7 Construction of the transformation system of *C. indicum.*

Supplemental Figure S8 Nucleotides and deduced amino acid sequence alignment of CiRhaT-SX_{2x} and CiRhaT-JS_{2x}.

Supplemental Figure S9 Silencing effects of *CiRhaT-*GD_{4x} gene in transgenic *Chrysanthemum.*

Supplemental Table S1 Parameters of linear regression and experimental retention times (t_R), LOD, LOQ, and RSD (%) for the studied compounds through LC-MS/MS.

Supplemental Table S2 Amino acid sequences of CiRhaT-GD_{4x} and some known 1,6-RhaTs were aligned and analyzed using Clustal W.

Supplemental Table S3 List of 1,6/1,2 glycosidic bond flavonoid UGTs used in the phylogenetic analysis.

Supplemental Table S4 Nonsynonymous substitution of RhaT sequences in *Chrysanthemum*.

Supplemental Table S5 For clone, vector and RT-qPCR primers used in this study. Restriction enzyme sites are highlighted by bold and underline format.

Supplemental Figure S1 Extracted-ion chromatogram (XIC) of the methanol extracts of *Chrysanthemum* plants. Ion chromatograms corresponding to acacetin-7-*O*-glucoside, linarin (acacetin-7-*O*-rutinoside), apigenin-7-*O*-glucoside, isorhoifolin (apigenin-7-*O*-rutinoside), diosmetin-7-*O*-glucoside, and diosmin (diosmetin-7-*O*-rutinoside) were extracted. The selected *Chrysanthemum indicum*$_{2x}$ population included **A** Hubei Province, **B** Anhui Province, **C** Shanxi Province, **D** *Chrysanthemum indicum*$_{4x}$ from Guangdong Province, **E** Jiangxi Province, **F** Hubei Province; *Chrysanthmum nankingense*$_{2x}$ population from **G** Jiangsu Province, and **H** Hubei Province.

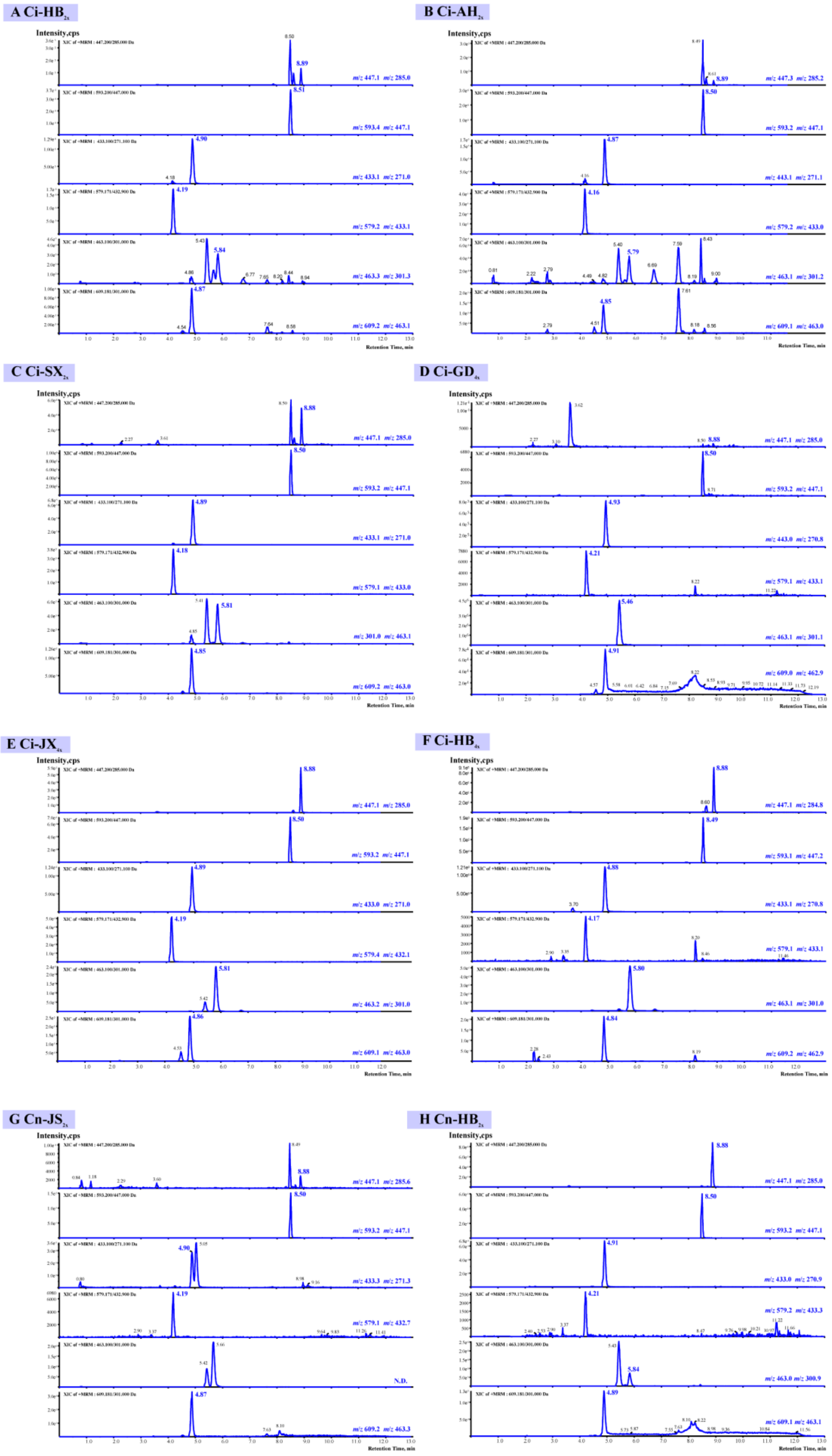
A Ci-HB$_{2s}$
B Ci-AH$_{2s}$
C Ci-SX$_{2s}$
D Ci-GD$_{4s}$
E Ci-JX$_{4s}$
F Ci-HB$_{4s}$
G Cn-JS$_{2s}$
H Cn-HB$_{2s}$
Intensity,cps
Retention Time, min
N.D.

Supplemental Figure S2 RT-qPCR analysis of *RhaT* transcripts in different tissues and organs and subcellular localization. **A**, Transcript abundance of *CiRhaT-GD*$_{4x}$ in different tissues. *EF1α* was selected as an internal control, and asterisks indicate significant differences in comparisons among the values of *CiRhaT-GD*$_{4x}$ transcripts in Root. Asterisks indicate significant differences (**, $P < 0.01$, ***, $P < 0.001$) analyzed through one-way ANOVA with LSD multiple comparison test. **B**, Relative expression level of *RhaTs* in different ploidy levels. Significant difference ($P < 0.001$) is indicated by asterisk (***) compared with the CiRhaT-GD$_{4x}$ group by using Student's *t* test. For (**A**) and (**B**), the error bars represent standard deviations, derived from three biological repeats, each with three technical repeats. **C**, Subcellular localization of CiRhaT-GD$_{4x}$ in *Nicotiana benthamiana* leaves, and the empty vector pCG3301 with the GFP tag was used as control. Scale bar: 30 μm.

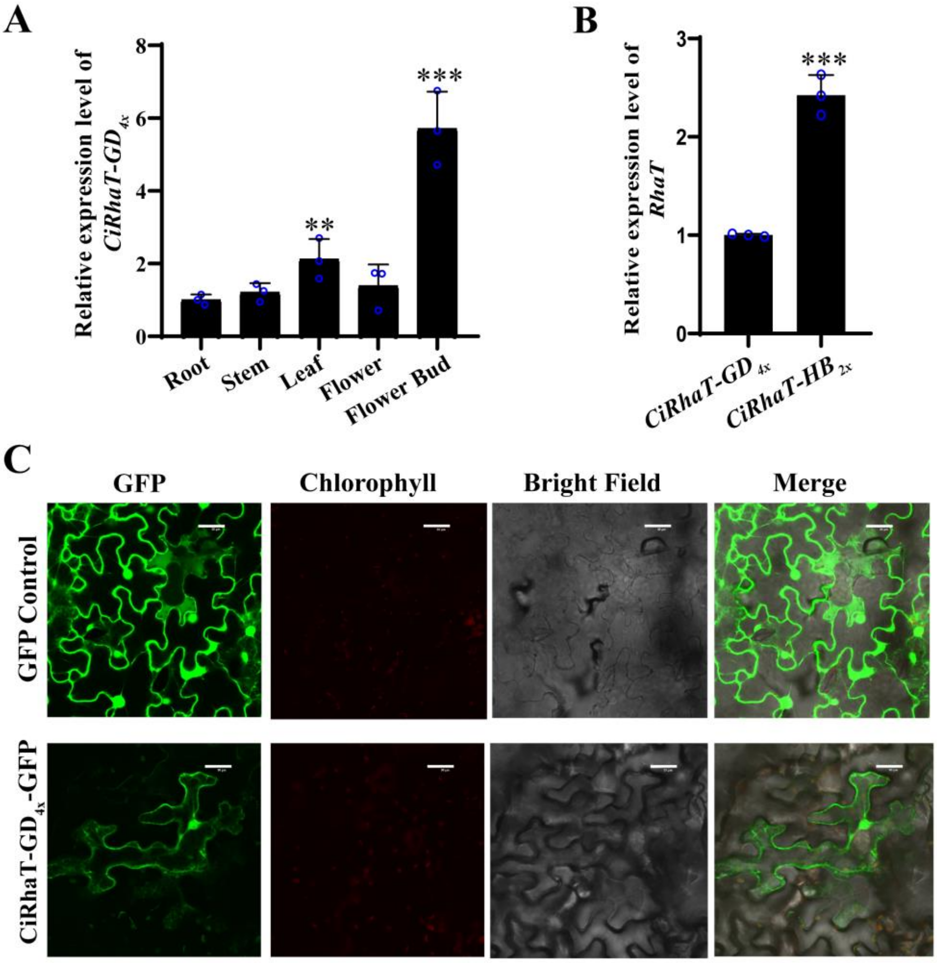

Supplemental Figure S3 Amino acid alignment of *Chrysanthemum* and *Citrus* RhaTs. *Dashes* represent gaps introduced to improve the alignment. The percentage identify threshold was 30% or above. The plant secondary product glycosyltransferase (PSPG) box is in *red*. The sequences of *Citrus* RhaTs downloaded from GeneBank were aligned using Jalview software.

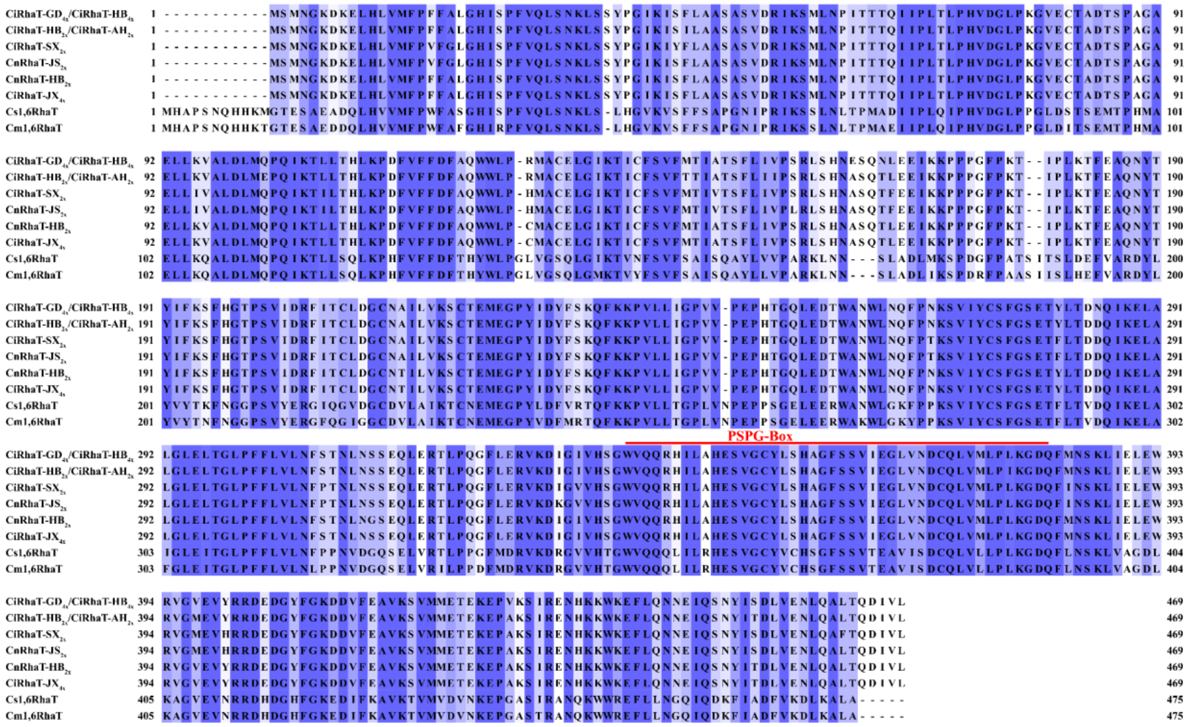

Supplemental Figure S4 Expression of recombinant RhaT enzymes in *E. coli*. All MBP-tagged proteins were purified using a dextrin bead 6FF column. SDS-PAGE indicated that enzymes were well expressed in *E. coli* Rosetta (DE3). Lanes M, molecular weight standards; the molecular mass of the standards is shown. Proteins were separated on a 10% SDS–polyacrylamide gel.

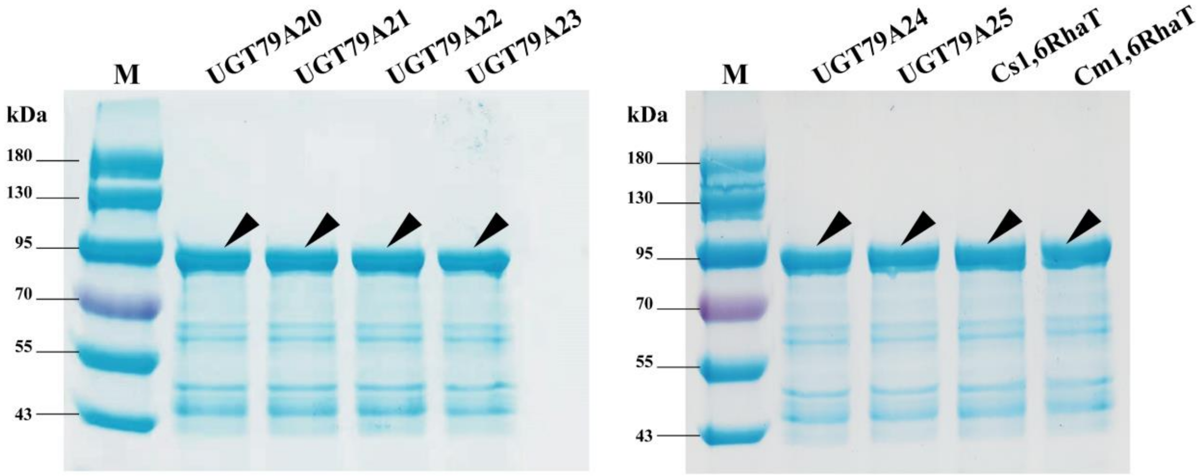

Supplemental Figure S5 Standard curves of seven flavonoid rutinoside compounds in determination of relative activity. **A**, Acacetin-7-*O*-rutinoside (**1a**). **B**, Apigenin-7-*O*-rutinoside (**2b**). **C**, Diosmetin-7-*O*-rutinoside (**3a**). **D**, Naringenin-7-*O*-rutinoside (**4a**). **E**, Hesperetin-7-*O*-rutinoside (**5a**). **F**, Quercetin-3-*O*-rutinoside (**6a**). **G**, Kaempferol-3-*O*-rutinoside (**7a**).

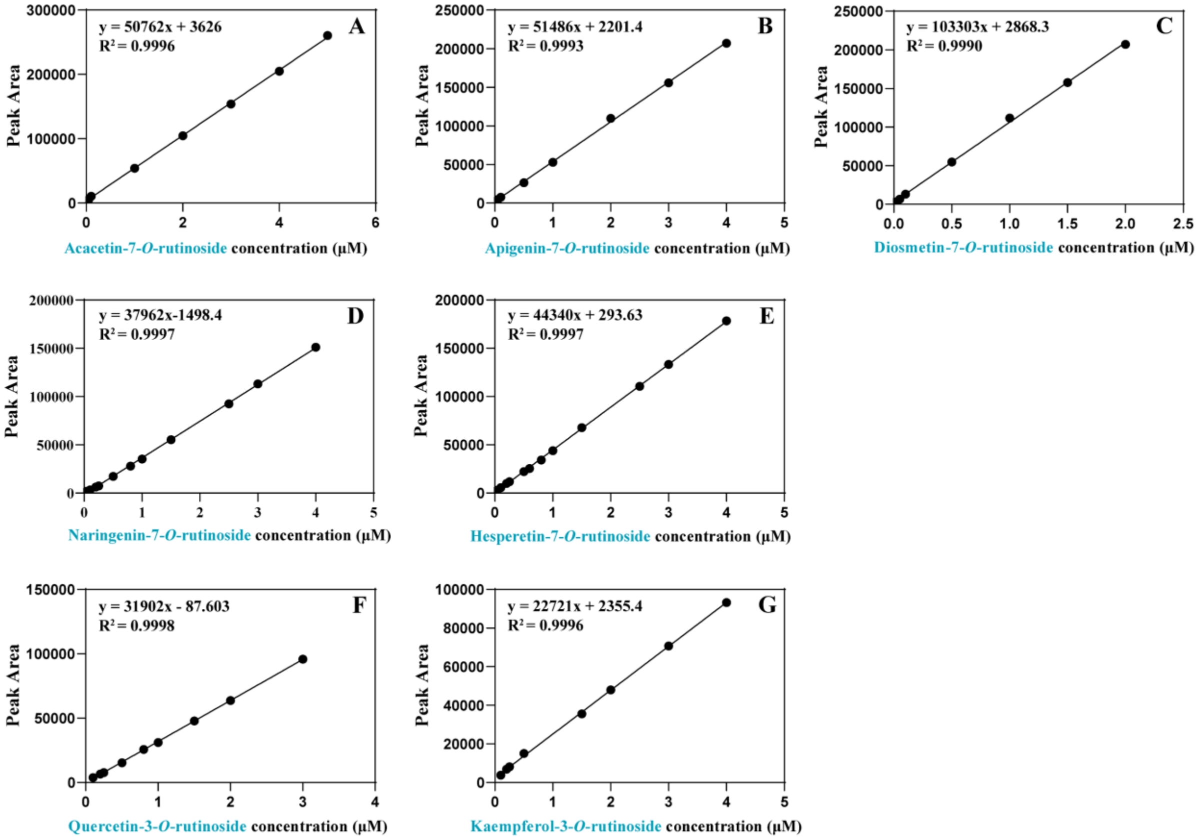

Supplemental Figure S6 Regeneration and culture of *C. indicum* hairy roots after infection with *A. rhizogenes*. **A**, Explants 14 days after infection with the 15-day-old seedling of *C. indicum*. **B**, Hairy roots after 21-day culture. **C**, 46 days culture in MS liquid medium. **D**, Genomic PCR analysis of genes in transformed hairy roots. Lane M: marker 2000 bp, Lane Rol A and Rol B as the positive control, Lane 1: Hyg primer, Lane 2: GFP primer, Lane 3: CiRhaT primer, Lane 4-6: Universal and specific primers.

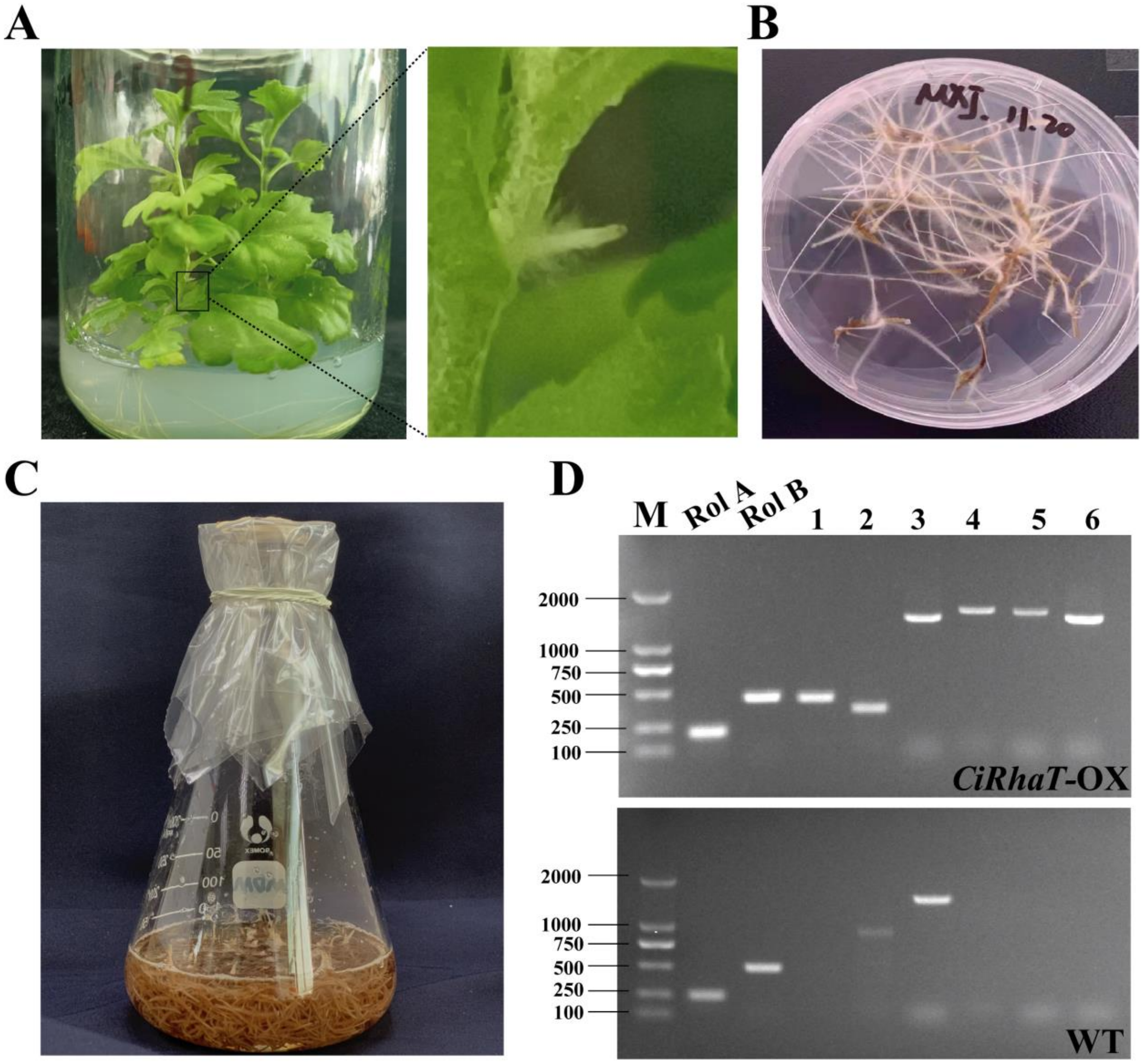

Supplemental Figure S7 Construction of the transformation system of *C. indicum.* **A**, Using axillary nodes as explants for culture. **B**, The callus proliferated and differentiated in numerous adventitious buds. **C**, Nonpositive adventitious buds appear whitening. **D**, Resistant plants. **E**, Positive transgenic lines were identified through genomic PCR.

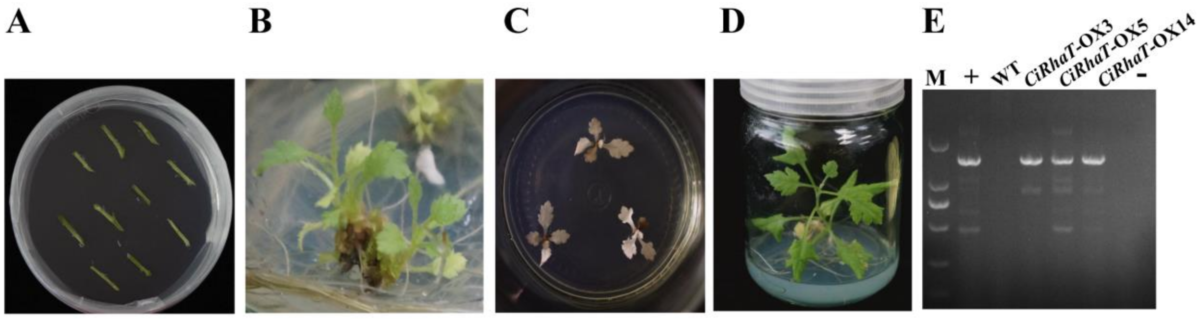

Supplemental Figure S8 Nucleotides and deduced amino acid sequence alignment of CiRhaT-SX$_{2x}$ and CiRhaT-JS$_{2x}$. Sequence differences are highlighted in blue. **A**, The amino acid sequence alignment. **B**, The nucleotides sequence alignment.

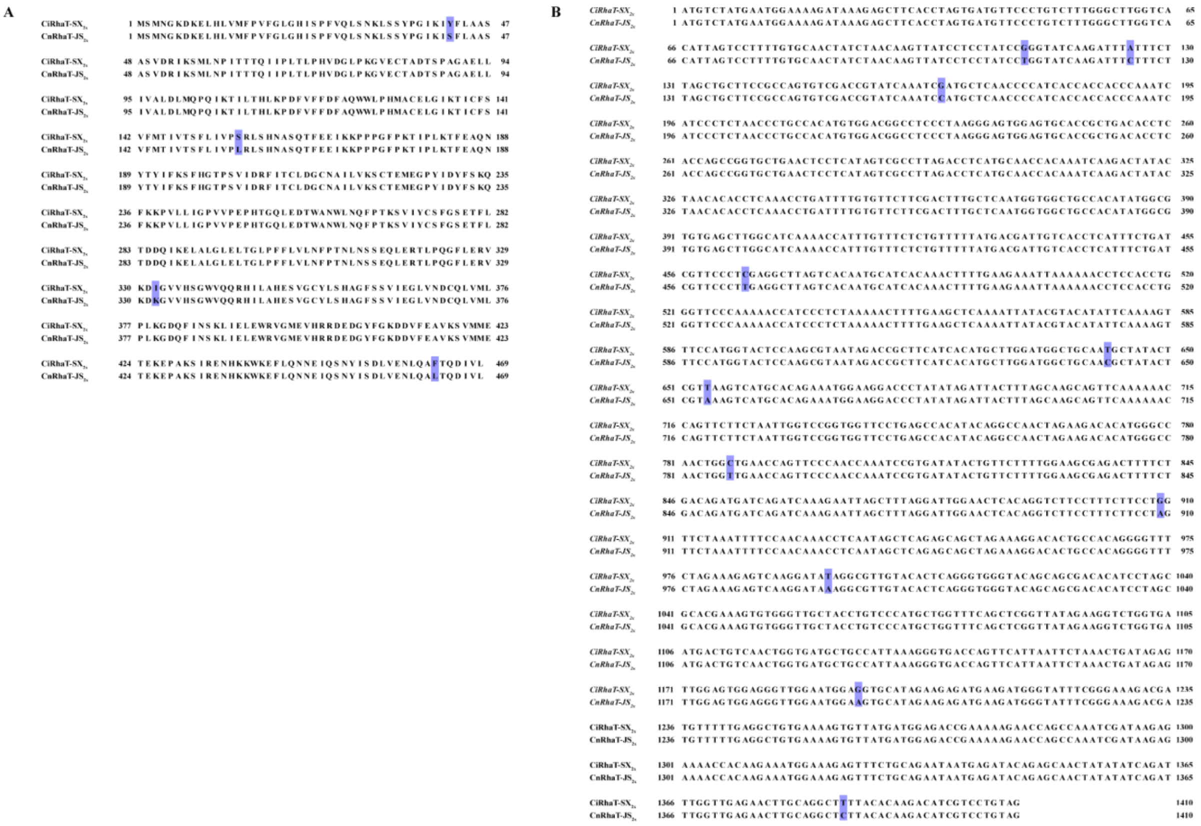

Supplemental Figure S9 **S**ilencing effects of *CiRhaT-GD*$_{4x}$ gene in transgenic *Chrysanthemum*. **A**, Schematic diagram of pRNAi-*CiRhaT-GD*$_{4x}$ constructs used for *Chrysanthemum* transformation. **B**, Albino phenotype of *CiRhaT*-silenced lines.

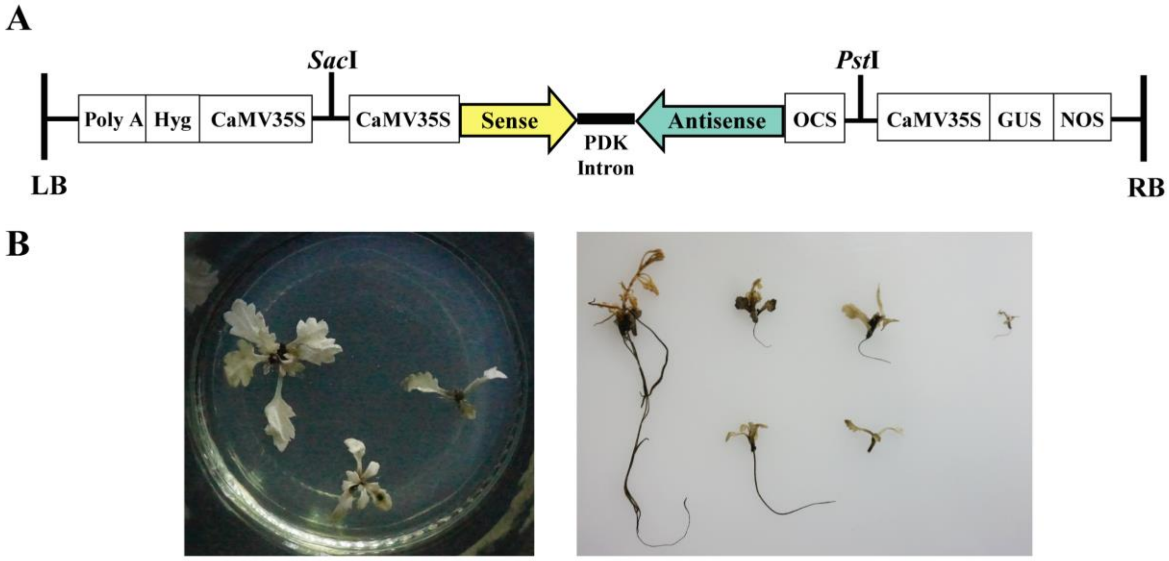

Supplemental Table S1 Parameters of linear regression and experimental retention times (t_R), LOD, LOQ, and RSD (%) for the studied compounds through LC-MS/MS.

Compounds	Retention time (min)	R^2	Linear range (μg/mL)	Regression equation	LOD (μg/mL)	LOQ (μg/mL)	RSD (%)
Acacetin-7-*O*-glucoside (**1**)	8.88±0.02	0.99895	0.017856-0.8928	$y=3.06275e^{6} x -8708.09248$	0.000008928	0.00004464	3.16
		0.99907	0.00017856-0.017856	$y = 3.38539e^{6} x + 451.76432$			
Acacetin-7-*O*-rutinoside (**1a**)	8.50±0.01	0.99936	1.185-11.85	$y = 8.51746e^{5} x + 8.04575e^{5}$	0.000237	0.001185	2.05
		0.99999	0.00237-1.185	$y = 1.37577e^{6} x + 3572.41117$			
Apigenin-7-*O*-glucoside (**2**)	4.90±0.02	0.99915	0.1801-5.405	$y = 3.91964e^{6} x + 4.58598e^{5}$	0.0001081	0.0002162	1.29
		0.99971	0.0005405-0.1081	$y = 5.56283e^{6} x + 374.60588$			
Apigenin-7-*O*-rutinoside (**2a**)	4.18±0.03	0.99906	0.02314-1.157	$y = 5.93105e^{6} x + 17208.44534$	0.00001157	0.0001157	1.77
		0.99997	0.0002314-0.02314	$y = 6.68738e^{6} x -338.74374$			
Diosmetin-7-*O*-glucoside (**3**)	5.83±0.03	0.99952	0.018496-0.9248	$y = 3.22283e^{7} x + 6.79822e^{5}$	0.000018496	0.0002	2.72
		0.99985	0.00009248-0.018496	$y = 4.20957e^{7} x + 131.00427$			
Diosmetin-7-*O*-rutinoside (**3a**)	4.86±0.03	0.99916	0.00122-0.6085	$y = 1.41489e^{7} x + 17856.49302$	0.000998	0.00122	12.52

Supplemental Table S2 Amino acid sequences of CiRhaT-GD$_{4x}$ and some known 1,6-RhaTs were aligned and analyzed using Clustal W.

CiRhaTs-GD$_{4x}$	Unigene ID	Amino acid sequence identity (%)					Gene Length
		Cs1,6RhaT	Cm1,6RhaT	Gm1,6RhaT	Fe1,6RhaT	Ph1,6RhaT	
CiRhaT1-GD$_{4x}$	Unigene0007998	55.22	52.88	51.29	54.84	60.13	1410
CiRhaT2-GD$_{4x}$	Unigene0014370	24.12	23.67	25.22	24.78	25.44	1359
CiRhaT3-GD$_{4x}$	Unigene0018680	26.11	26.11	26.94	27.74	27.70	1431
CiRhaT4-GD$_{4x}$	Unigene0044445	18.60	18.16	21.88	21.66	21.44	1374
CiRhaT5-GD$_{4x}$	Unigene0059003	27.39	27.61	26.30	28.48	26.52	1383

Supplemental Table S3 List of 1,6/1,2 glycosidic bond flavonoid UGTs used in the phylogenetic analysis.

**Name**	**Function**	**Organism**	**Accession No.**[a]
Ph1,6RhaT (UGT79A1)	Anthocyanidin 3-*O*-glucoside 6″-*O*-rhamnosyltransferase	*Petunia x hybrida*	X71059
Gm1,6GlcT (UGT79A7)	Flavonol 3-*O*-glucoside 6″-*O*-glucosyltransferase	*Glycine max*	LC126028
Gm1,6RhaT (UGT79A6)	Flavonol 3-*O*-glucoside 6″-*O*-rhamnosyltransferase	*Glycine max*	AB828193
Cs1,6RhaT	Flavanone 7-*O*-glucoside 6″-O-rhamnosyltransferase	*Citrus sinensis*	DQ119035
Cm1,6RhaT	Flavanone 7-*O*-glucoside 6″-*O*-rhamnosyltransferase	*Citrus maxima*	LC057678
Fe1,6RhaT (UGT79A8)	Flavonol 3-*O*-glucoside 6″-*O*-rhamnosyltransferase	*Fagopyrum esculentum*	LC312144
At1,2GlcT (UGT79B6)	Flavonoid 3-*O*-glucoside 2″-*O*-glucosyltransferase	*Arabidopsis thaliana*	NM_124780
At1,2XylT (UGT79B1)	Anthocyanin 3-*O*-glucoside 2″-*O*-xylosyltransferase	*Arabidopsis thaliana*	NM_124785
Gm1,2GlcT (UGT79B30)	Flavonol 3-*O*-glucoside 2″-*O*-glucosyltransferase	*Glycine max*	LC017844
Ip1,2GlcT (UGT79B16)	Anthocyanidin 3-*O*-glucoside 2″-*O*-glucosyltransferase	*Ipomoea purpurea*	AB192315
In1,2GlcT	Anthocyanidin 3-*O*-glucoside 2″-*O*-glucosyltransferase	*Ipomoea nil*	AB192314
Bp1,2GAT (UGT94B1)	Anthocyanin 3-*O*-glucoside 2″-*O*-glucuronosyltransferase	*Bellis perennis*	AB190262
Cm1,2RhaT	Flavanone 7-*O*-glucoside 2″-*O*-rhamnosyltransferase	*Citrus maxima*	AY048882

[a]Accession number in NCBI (http://www.ncbi.nlm.nih.gov/).

Supplemental Table S4 Nonsynonymous substitution of RhaT sequences in *Chrysanthemum*.

RhaTs	17	42	43	95	102	108	128	144	147	153	155	161	164	215	260
CiRhaT-GD$_{4x}$	F	S	F	K	Q	L	R	M	A	V	S	E	N	A	A
CiRhaT-HB$_{4x}$	F	S	F	K	Q	L	R	M	A	V	S	E	N	A	A
CiRhaT-JX$_{4x}$	F	S	F	K	Q	L	C	M	A	V	S	A	T	T	A
CiRhaT-HB$_{2x}$	F	S	I	K	E	L	R	T	A	I	S	A	T	A	A
CiRhaT-AH$_{2x}$	F	S	I	K	E	L	R	T	A	I	S	A	T	A	A
CiRhaT-SX$_{2x}$	V	Y	F	I	Q	I	H	M	V	V	S	A	T	A	A
CnRhaT-HB$_{2x}$	F	S	F	K	Q	L	C	M	A	V	S	A	T	T	A
CnRhaT-JS$_{2x}$	V	S	F	I	Q	I	H	M	V	V	L	A	T	A	A

RhaTs	268	281	285	308	313	332	334	378	384	390	397	400	429	454	463
CiRhaT-GD$_{4x}$	N	Y	N	S	S	I	I	L	M	E	V	Y	V	S	L
CiRhaT-HB$_{4x}$	N	Y	N	S	S	I	I	L	M	E	V	Y	V	S	L
CiRhaT-JX$_{4x}$	N	Y	D	S	S	I	I	L	M	E	V	Y	A	T	L
CiRhaT-HB$_{2x}$	N	Y	D	S	S	I	I	I	M	E	M	Y	A	T	L
CiRhaT-AH$_{2x}$	N	Y	D	S	S	I	I	I	M	E	M	Y	A	T	L
CiRhaT-SX$_{2x}$	T	F	D	P	S	I	V	L	I	E	M	H	A	S	F
CnRhaT-HB$_{2x}$	N	Y	D	S	G	I	I	L	M	E	V	Y	A	T	L
CnRhaT-JS$_{2x}$	T	F	D	P	S	K	V	L	I	E	M	H	A	S	L

Supplemental Table S5 For clone, vector and RT-qPCR primers used in this study. Restriction enzyme sites are highlighted by bold and underline format.

Primer	Sequence (5′→3′)
CiRhaT1-GD$_{4x}$-F	ATGTCTATGAATGGAAAAGATAAAGAGC
CiRhaT1-GD$_{4x}$-R	CTACAGGACGATGTCTTGTGTAAGAG
CiRhaT2-GD$_{4x}$-F	ATGGATTCAAAAAACGAGAATTCAAG
CiRhaT2-GD$_{4x}$-R	TCATTTTTTTGGTTCACATAGCTTGACC
CiRhaT3-GD$_{4x}$-F	ATGACTATCACAAACTCTATGGACAA
CiRhaT3-GD$_{4x}$-R	TTAACAATCGACTGTCTCTTGCTTC
CiRhaT4-GD$_{4x}$-F	ATGGATTCAACAAATAATAGATATCG
CiRhaT4-GD$_{4x}$-R	CTAATTAAAATTCTTCAGTTGACAAAG
CiRhaT5-GD$_{4x}$-F	ATGGCAAAAAATGGAGATGGTAGTCT
CiRhaT5-GD$_{4x}$-R	CTATTTTGAGGAGTTTTGAAGAAAATGC
pMAL-c5X-CiRhaT1-GD$_{4x}$-F	GAAGGATTTCA**CATATG**ATGTCTATGAATGGAAAAGATAAAGAGCTTCACC
pMAL-c5X-CiRhaT1-GD$_{4x}$-R	GTTTTATTTG**AAGCTT**CTACAGGACGATGTCTTGTGTAAGAGC
pMAL-c5X-CiRhaT2-GD$_{4x}$-F	GAAGGATTTCA**CATATG**ATGGATTCAAAAAACGAGAATTCAAGATATCGT
pMAL-c5X-CiRhaT2-GD$_{4x}$-R	GTTTTATTTG**AAGCTT**TCATTTTTTTGGTTCACATAGCTTGACCAAAC
pMAL-c5X-CiRhaT3-GD$_{4x}$-F	GAAGGATTTCA**CATATG**ATGACTATCACAAACTCTATGGACAAACGT
pMAL-c5X-CiRhaT3-GD$_{4x}$-R	GTTTTATTTG**AAGCTT**TTAACAATCGACTGTCTCTTGCTTCTCC

pMAL-c5X-CiRhaT4-GD$_{4x}$-F	GAAGGATTTCA**<u>CATATG</u>**ATGGATTCAACAAATAATAGATATCGTATAATATTGTTGCCA
pMAL-c5X-CiRhaT4-GD$_{4x}$-R	GTTTTATTTG**<u>AAGCTT</u>**CTAATTAAAATTCTTCAGTTGACAAAGCTTGACTAAATTCT
pMAL-c5X-CiRhaT5-GD$_{4x}$-F	GAAGGATTTCA**<u>CATATG</u>**ATGGCAAAAAATGGAGATGGTAGTCTT
pMAL-c5X-CiRhaT5-GD$_{4x}$-R	GTTTTATTTG**<u>AAGCTT</u>**CTATTTTGAGGAGTTTTGAAGAAAATGCAGCA
pCAMBIA1302-RhaT-F	TGGAGAGAACACGGGGGACTCTTGA**<u>CCATGG</u>**ATGTCTATGAATGGAAAAGATAAAG
pCAMBIA1302-RhaT-R	CTCCAGTGAAAAGTTCTTCTCCTTT**<u>ACTAGT</u>**CTACAGGACGATGTCTTGTGTAAGA
CaMV35S-F	CACTGACGTAAGGGATGACGCACAAT
1302-GFP-R	CCATCTAATTCAACAAGAATTGGGACAAC
RT-qPCR-CiRhaT-GD$_{4x}$-OX-F	GCCAGTGTCGACCGTATCAA
RT-qPCR-CiRhaT-GD$_{4x}$-OX-R	AGGCGACTTTGAGGAGTTCAG
EF1a-F	GGTCAGATTGGAAACGGTTAT
EF1a-R	AGGTGGGTATTCAGCAAAGG
RT-qPCR-CiRhaT-GD$_{4x}$-F	AAAGGACACTGCCACAAGGG
RT-qPCR-CiRhaT-GD$_{4x}$-R	CAGGTAGCACCCCACACTTT
Rol A-F	GCTCGTTGTCTCCGACCTAT
Rol A-R	GGTCTGAATATTCCGGTCCA
Rol B-F	GCCAGCATTTTTGGTGAACT
Rol B-R	CTGGCCCATCGTTCTAAAAA
Hyg-F	GGCGACCTCGTATTGGGAAT
Hyg-R	TGACCTATTGCATCTCCCGC
GFP-F	TACGTGCAGGAGAGGACCAT

GFP-R	CGAAAGGGCAGATTGTGTGG
pCG3301-CiRhaT-GD$_{4x}$-F	AGAACACGGGGGAC**GAGCTC**ATGTCTATGAATGGAAAAGATAAAGAGCTTCACC
pCG3301-CiRhaT-GD$_{4x}$-R	TCACCATGGT**GTCGAC**CAGGACGATGTCTTGTGTAAGAGC
